# Gasdermin-B Promotes Invasion and Metastasis in Breast Cancer Cells

**DOI:** 10.1371/journal.pone.0090099

**Published:** 2014-03-27

**Authors:** Marta Hergueta-Redondo, David Sarrió, Ángela Molina-Crespo, Diego Megias, Alba Mota, Alejandro Rojo-Sebastian, Pablo García-Sanz, Saleta Morales, Sandra Abril, Amparo Cano, Héctor Peinado, Gema Moreno-Bueno

**Affiliations:** 1 Departamento de Bioquímica, Universidad Autónoma de Madrid (UAM), Instituto de Investigaciones Biomédicas "Alberto Sols" (CSIC-UAM), IdiPAZ, Madrid, Spain; 2 Centro Nacional de Investigaciones Oncológicas, CNIO, Madrid, Spain; 3 Hospital MD Anderson Cancer Centre, Madrid, Spain; 4 Fundación MD Anderson Internacional, Madrid, Spain; 5 Children's Cancer and Blood Foundation Laboratories, Departments of Pediatrics, Cell and Developmental Biology, Weill Cornell Medical College, New York, New York, United States of America; Institute of Molecular and Cell Biology, Biopolis, United States of America

## Abstract

Gasdermin B (GSDMB) belongs to the Gasdermin protein family that comprises four members (GSDMA-D). Gasdermin B expression has been detected in some tumor types such as hepatocarcinomas, gastric and cervix cancers; and its over-expression has been related to tumor progression. At least four splicing isoforms of GSDMB have been identified, which may play differential roles in cancer. However, the implication of GSDMB in carcinogenesis and tumor progression is not well understood. Here, we uncover for the first time the functional implication of GSDMB in breast cancer. Our data shows that high levels of GSDMB expression is correlated with reduced survival and increased metastasis in breast cancer patients included in an expression dataset (>1,000 cases). We demonstrate that *GSDMB* is upregulated in breast carcinomas compared to normal breast tissue, being the isoform 2 (GSDMB-2) the most differentially expressed. In order to evaluate the functional role of GSDMB in breast cancer two GSDMB isoforms were studied (GSDMB-1 and GSDMB-2). The overexpression of both isoforms in the MCF7 breast carcinoma cell line promotes cell motility and invasion, while its silencing in HCC1954 breast carcinoma cells decreases the migratory and invasive phenotype. Importantly, we demonstrate that both isoforms have a differential role on the activation of Rac-1 and Cdc-42 Rho-GTPases. Moreover, our data support that GSMDB-2 induces a pro-tumorigenic and pro-metastatic behavior in mouse xenograft models as compared to GSDMB-1. Finally, we observed that although both GSDMB isoforms interact *in vitro* with the chaperone Hsp90, only the GSDMB-2 isoform relies on this chaperone for its stability. Taken together, our results provide for the first time evidences that GSDMB-2 induces invasion, tumor progression and metastasis in MCF7 cells and that GSDMB can be considered as a new potential prognostic marker in breast cancer.

## Introduction

Gasdermin protein superfamily (PF04598) is constituted of eight structurally-related genes in the mouse (Gsdma1, Gsdma2, Gsdma3, Gsdmc1, Gsdmc2, Gsdmc3, Gsdmc4, Gsdmd), and four genes in human: *Gasdermin A* (GSDMA), *Gasdermin B (GSDMB)*, *Gasdermin C (GSDMC)* and *Gasdermin D (GSDMD)* [1]–[11]. *GSDMB* (previously known as *PRO2521*, *GSDML*) seems to have originated from a duplication of *GSDMA* gene during the evolution of this gene family, being the only GSDM member not present in the rodent genome [10]. The identification of mouse *Gsdma3* as the gene responsible for an abnormal skin phenotype (epidermal hyperplasia, hyperkeratosis, and abnormal hair development) of two mutant mice led to the characterization of the *Gsdm* gene family [1], [2]. *Gsdm* genes have a tissue-specific expression pattern in gastric epithelia and epidermis, suggesting that they may contribute to the regulation of normal epithelial cell proliferation and /or differentiation [11]. However, there is scarce information about the expression pattern of human *GSDM* genes.

Although the four human proteins of this family contain several conserved sequences in the N- and C- terminal regions, to date no functional domains or motifs have been described. Consequently, the biological function of these proteins in physiological and pathological situations is still largely unknown. Recently, genetic polymorphisms in the *loci* containing *GSDMB* and *GSDMA* genes have been correlated with childhood asthma susceptibility [12], but the potential functional role of these genes in this pathology remains to be uncovered.

Interestingly, the altered expression of *GSDM* genes has been also associated to cancer. *GSDMA* is frequently found down-regulated in human gastric and skin cancer tissues and cancer-derived cell lines [4], [5]. Furthermore, GSDMA is involved in the TGF-beta signaling mediating the apoptotic activity in the gastric epithelium [4]. In contrast, *GSDMC* over-expression is associated with an increase in the metastatic potential in melanoma cell lines [6] and *GSDMD* expression is observed in the majority of gastric cancers [5].

*GSDMB* expression has been described in human gastric, liver and colon cancer cell lines and carcinomas, as well as in normal tissues [7]. *GSDMB* over-expression has been described in gastric and cervical tumors compared with normal tissue and this alteration is associated to tumor progression [7], [8]. GSDMB is located in the same chromosomal region than GSDMA; however, their expression is neither overlapping nor complementary during cancer development and progression [9]. The comparative analysis of these proteins suggests that *GSDMA* may act as tumor suppressor gene in gastric cancer, while *GSDMB* could be considered as an oncogene based on its amplification and over-expression in this cancer type [5]. Although *GSDMB* expression has been reported in the secretory cells in gastric and hepatic carcinomas [7], there are some discrepancies in its expression pattern depending on the tissue or cell system analyzed [7], [8]. There are also evidences that *GSDMB* presents different splicing variants that may have differential effects on tumor growth and development [7], [8]. Four different isoforms have been described, which differ in exons 6 and 7 of the *GSDMB* gene (Figure S1) [7], [8]. However, the relevance and specific functional role of these variants in cancer is still unknown.

Based on these data, we investigated the potential role of GSDMB in breast cancer. The analysis of *GSDMB* expression in a dataset of more than 1,000 human breast cancer tumors reveals that high levels of expression are correlated with reduced survival and increased metastasis. Our *in vitro* analysis in MCF7 cells over-expressing *GSDMB-1* and -*2* isoforms reveal common and distinct functions of these GSDMB variants in breast cancer progression. While both GSDMB isoforms promote cell motility and invasion through activation of the Rho-GTPases Rac-1 and Cdc-42 *in vitro*, their analysis in xenograft mouse models showed that only GSDMB-2 increases tumor growth and metastasis. Moreover, silencing the endogenous GSDMB in HCC1954 breast carcinoma cells reduces their migratory and invasive capacity. Finally, we report that GSDMB-2 is a novel client protein of Hsp90, since its stability relies on this chaperone. Our data indicate for the first time that GSDMB, and specifically the isoform 2, is a new marker of breast cancer progression and a potential therapeutic target in order to block tumor growth and cell dissemination.

## Materials and Methods

### Breast samples

We studied a series of 18 sporadic ductal breast carcinomas, (6 of them classified as grade 1, 7 as grade 2 and the rest as grade 3) as well as 4 normal breast samples obtained from the Biobank of the MD Anderson Cancer Center Madrid, Spain. Patients underwent surgery between 2011 and 2012. The mean patient age at surgery was 57.3 years (range, 45 to 81 years). This study was performed following standard ethical procedures of the Spanish regulation (Ley de Investigación Orgánica Biomédica, 14 July 2007) and was approved by the ethical committee of the MD Anderson Cancer Center Madrid, Madrid, Spain.

### Cloning human GSDMB

Universal Human Reference RNA (Stratagene) was used for isolating *GSDMB* cDNA. First strand cDNA was made using Superscript II reverse transcriptase (Invitrogen) with oligo dT. The full-length coding regions of GSDMB-1 (NM_001042471.1) and GSDMB-2 (NM_018530.2) were amplified by PCR using gene-specific primers (Forward: 5′-GGGGGATCCATGTTCAGCGTATTTGAGGAAATC-3′; and Reverse: 5′-GCCTACCTCTGTCTCTTCCCTCGAGGGG-3′. Amplification reactions consisted of the following steps: 95°C for 5 min, 35 cycles at 95°C for 30 sec; 55°C for 1.5 min and 72°C for 10 min. The coding regions of both isoforms were cloned into the *Bam* HI and *Xho* I sites of plasmid pcDNA3-HA (Invitrogen). Full-length cDNA of human GSDMB-1 and -2 subtypes were confirmed by restriction mapping and DNA sequencing.

### Cell culture and reagents

Human carcinoma cell lines, MCF7, MDA-231, CAMA-1, T47D, HCC1954, HEK293T (human embryonic kidney 293 transformed with T-antigen) and non-tumorigenic breast cell line MCF10-2A were obtained from the American Type Cell Culture (ATCC) (LGC Standards-SLU) and cultured according to the indicated supplier conditions. Cell lines were authenticated using STR-profiling according to ATCC guidelines. Cells were maintained as monolayer cultures at 37°C in an atmosphere with 5% CO2. For generation of MCF7 over-expressing GSDMB-1 and -2 GSDMB variants the indicated cell lines were transfected using Lipofectamine (Invitrogen) with pcDNA3-GSDMB-1 (MCF7-G1) and -2 (MCF7-G2) tagged with hemagglutinin epitope (HA) at the C-terminal respectively, and independent clones were isolated with cloning rings in the presence of G418 (400 μg/ml) for 3-4 weeks. Control cells (MCF7-C) were obtained by stable transfection of empty pcDNA3-HA vector. At least 10 independent clones were isolated from each transfection. Two independent clones from each transfection were analysed and most representative results of one single clone are shown in the figures. MCF7 cells expressing mCherry (MCF7-mCherry) or firefly luciferase cells (MCF-Luc) were obtained by lentiviral infection with PRRL-cPTT-PGK-mCherry-W or GFP-luc viral particles (Gentarget Inc) respectively.

GSDMB silenced HCC1954 cells were obtained using Mission shRNA Lentiviral Transduction Particles (SIGMA-Aldrich). Two GSDMB shRNA sequences were validated: TRCN0000137108 (sh108) and TRCN0000168794 (sh794). Non-targeting Control shRNA Transduction Particles (SHC002V) were used as control.

The Hsp90 inhibitor 17-AAG (Sigma) was dissolved in DMSO to a stock concentration of 1 mM. MCF7 control, GSDMB-1 and GSDMB-2 over-expressing cells were treated with different concentration of 17-AAG (1,000, 500, 100, 50 nmol/L) or DMSO for 24 hours. Lysates were blotted with rabbit anti-Hsp90 (Cell Signalling), rat anti-HA (Roche) or mouse anti-tubulin (Sigma) antibodies.

The Rac1 inhibitor NSC23766 (TOCRIS) was dissolved in distilled water to a stock concentration of 100 mM. Migration assays were performed using modified polycarbonate nucleopore membranes (6.5 mm in diameter, 8-μm pore size) (Corning, USA). Cells (1×10^5^) were seeded on the upper part of each chamber in the presence of this inhibitor (100 μmol/L), and after incubation for 48–72 h, non-migrating cells on the upper surface of the filter were wiped with a cotton swab, and migrated cells on the lower surface of the filter were fixed, stained with DAPI, and counted by examination of at least six microscopic fields.

### Semiquantitative and quantitative RT-PCR analysis

Total RNA from cell lines and tumor samples was extracted with Trizol (Invitrogen) and RNaesy Extraction Kit (QIAGen) as indicated by the manufacturer. cDNA from the different cell lines and tumor samples was obtained from 1 μg of total RNA using random primers and Superscript II system (Life Technologies Inc) as previously described [13]. Gene expression analyses were performed by semiquantitative RT-PCR (sqRT-PCR) and real time RT-PCR (qRT-PCR). For qRT-PCR pre-designed TaqMan probes (GSDMB, GSDMB-1, GSDMB-2, GSDMB-3&4) or SybrGreen PCR reagents (mCherry) (Sigma) were used on an iQ5 iCycler Realtime PCR Detection System (BioRad) using TaqMan “iQ Supermix” or “SYBR Green Supermix” (BioRad), according to the manufacturer's recommendations. GSDMB-3 and -4 were analyzed together as there is no commercially available Taqman probe to discriminate isoform 3. Primers sequences and amplification conditions for sqRT-PCR and for qRT-PCR are indicated in Tables S1 and S2, respectively. All RT-PCRs were performed in triplicates. Relative expression was normalized to β2 microglobulin, β-actin or GAPDH. The comparative threshold cycle (Ct) method was used to calculate the amplification factor as specified by the manufacturer.

### Barrier Migration Assay and Immunofluorescence

For barrier migration assay, cells were grown to confluence on 10 mm glass coverslips as previously described [24]. The barrier assay was performed incubating the coverslips on chambers (Lab-Tek, Nunc) and cells were cultured for at least 5 days at 37°C in an atmosphere with 5% CO_2_. For the immunofluorescence analysis the cells were fixed in 4% formaldehyde at room temperature for 15 minutes and permeabilized for 5 minutes using 0.5% Triton X 100 (Sigma). After washing, cells were incubating with Alexa-647–coupled phalloidin (Molecular Probes) to stain F-actin. Cell nuclei were stained using 4,6-diamidino-2-phenylindole (DAPI, Molecular Probes). For the rest of inmunofluorescence assays, after fixation, permeabilization and blocking (25×10^5^ cells/coverslip) cells were incubated with first and secondary antibodies for 1 hour at room temperature. Primary antibodies were: rat anti-HA 1∶250 (Roche); mouse anti-tubulin 1∶4000 (Sigma), and mouse anti-Rac1 1∶100 (BD Transduction). Secondary antibodies were goat anti-mouse (1∶1000), anti-rat (1∶5000) conjugated with Alexa-488, Alexa-594 or Alexa-647 (GE Molecular Probes). For tissue immunofluorescence, mice lungs tissues were fixed in a mix of 2% PFA and 20% sucrose overnight and cryo-embedded in Tissue Tek O.C.T. embedding compound. Sections (5 μm) were stained with DAPI and mCherry positive cells were detected by their intrinsic signal. In all cases fluorescent images were obtained using a Leica TCS SP5 confocal microscope (x63 objetive) and analyzed using the Leica LAS AF software. Digital images of mCherry stained sections were analyzed and pixels were quantified with ImageJ Software (NIH). Phase-contrast images of the indicated cells were taken using an inverted Zeiss Axiovert microscope.

### Invasion assay

Invasion assays of the indicated cell lines were performed using modified Boyden chambers with polycarbonate nucleopore membranes (Corning, USA). Filters (6.5 mm in diameter, 8-μm pore size) were coated with Matrigel as previously described [13], [14]. In brief, cells (1×10^5^) were seeded on the upper part of each chamber, and after incubation for 24 h, non-invading cells on the upper surface of the filter were wiped with a cotton swab, and migrated cells on the lower surface of the filter were fixed, stained with DAPI, and counted by examination of at least five microscopic fields.

### Fluorescent gelatin substrate degradation assay

Gelatin-FITC substrate (Invitrogen) was prepared as previously described [15]. Cells (25×10^3^) were placed on coverslips (Lab-Tek, nunc) previously covered with fluorescent gelatin substrate and incubated in RPMI with 10% fetal bovine serum (FBS) at 37°C in an atmosphere with 5% CO2 overnight. The cells were fixed and permeabilized with 4% paraformaldehyde and 0.05% Triton X-100 respectively for 5 min each, washed with PBS and actin was visualized by staining with Alexa Flour-647 Phalloidin (Invitrogen) for 30 min and nuclei were stained with DAPI followed by washes with PBS. Confocal microscopy analyses were performed using a Leica TCS SP5 confocal microscope, x63 objective. Degradation area was calculated by dividing the total area of the degraded zones per cell by the number of cells presents in each field using the ImageJ program.

### Gelatin Zymography

To evaluate the activity of MMP-2 and -9 activity cells were cultured in serum-free RPMI for 24 hours. Briefly, samples were prepared with standard SDS-gel-loading buffer containing 0.01% SDS without β-mercaptoethanol and heating. The samples were subjected to electrophoresis SDS-PAGE in 8% gels containing 0.1% gelatin. Following electrophoresis, the gels were washed in 2.5% Triton X-100 at room temperature to remove SDS, incubated in 100 mL reaction buffer (40 mM Tris-HCl, pH 8.0, 10 mM CaCl_2_, 0.02% NaN_3_) for 24 h at 37°C and stained with Coomassie brilliant blue R-250 containing 50% methanol and 10% acetic acid. Gelatinolytic activities were visualized by negative staining with 20% methanol and 10% acetic acid. All samples were analyzed in duplicate. Finally, the gels were scanned and subjected to densitometry analysis using Image J software. Relative density was calculated by dividing the intensity of the active MMP-9 band by the pro MMP-9 band, and then normalizing the data to the corresponding bands in the control cells.

### Proliferation assays

2.5×10^4^ cells were grown into 96-wells plate according to Cell Proliferation ELISA, BrdU (colorimetric) kit, (Roche Diagnostic SL, Basel, Switzerland) using the manufacturer's recommendations. Alternative, alamarBlue assay (Thermo Scientific) was performed according to the manufacturer's protocol to analyze the proliferation of shGSDMB and shControl- HCC1954 cells.

### Analysis of Rho-A, Rac-1 and Cdc-42 activity

To detect Rho-A-GTP, Rac-1 (Rac-1-GTP) and Cdc-42 (Cdc-42-GTP) in cell lysates, we used a Rho-A Activation Assay Kit (17-294) and Rac-1/Cdc-42 Activation Assay Kit (17-441, Millipore), using the manufacturer's instructions.

### Subcellular fractionation assay, Western Blotting and Immunoprecipitation

For subcellular distribution of endogenous GSDMB, HCC1954 cells were harvested and fractionated using the Subcelullar Protein Fractionation Kit according to manufacturer's protocol (Thermo Fisher, Rockford, IL). For Western blotting, cells were lysed and proteins were extracted using standard RIPA buffer. Protein concentration was determined by BCA protein assay (Pierce, Rockford, IL, USA), and equal amounts of proteins were loaded in SDS–PAGE in 6–12% gels. Polypeptides were transferred onto Immobilon-P (Millipore) nitrocellulose membranes, and nonspecific binding was blocked with 5% nonfat dry milk. Immunoblots were incubated with the indicated antibodies: rat polyclonal anti-HA (Clone 3F10, Roche), 1∶500; mouse monoclonal anti-tubulin (T9026, Sigma), 1∶10.000; anti-GAPDH (MAB374, Millipore), 1∶50.000; anti-GSDMB (3D8, Santa Cruz), 1∶250; anti-High Molecular Weight Cytokeratins (CKs 1, 5, 14,17) (clone 34BE12, DAKO), 1∶100; rabbit polyclonal anti-Hsp90,(C45G5,Cell Signalling), 1∶1000; anti-Calnexin, (C5C9, Cell Signaling), 1∶1000; anti-Trimethyl (Lys4) HistoneH3 (Mab07473, Millipore), 1∶1000; anti-Akt 1∶1000 (9272, Cell Signaling), and goat anti-Snail2 (G-18, Santa Cruz); 1∶250. Secondary antibodies were HRP-coupled sheep goat anti-rat (1∶10000), anti-mouse (1∶1000) or anti-rabbit (1∶5000) (Amersham). Bands were visualized using ECL chemiluminescence kit (Amersham), quantified by densitometric scanning and normalized to β-actin or α-tubulin expression.

For immunoprecipitation, cells were lysed in a buffer containing 10 mM Tris-HCl (pH 7.5), 150 mM NaCl, 5 mM EDTA, and 1 mM PMSF. 1 mg of protein containing lysate was incubated with appropriate antibody overnight and then with protein A/G-sepharose for 1 hr at 4°C. After washing the beads with the lysis buffer three times, the protein bound to the beads was detected by Western blotting.

### Mammary fat pad inoculation and intracardiac experimental metastasis model

For primary tumor induction and spontaneous metastasis assays, MCF7-C and GSDMB-1 and -2 mCherry or Luciferase positive cells were orthotopically injected (5×10^6^ in 0.1 ml serum free growth medium) into the left fifth mammary fat pad (mfp) of five 8-week female nu/nu mice (Charles River) for each experimental condition (mCherry and Luc systems) as described in [14]. Tumor growth was measured once per week by determination of the two orthogonal external diameters using a calliper. Volumes were calculated using the formula (4π/3)xL xW^2^, where L and W are the length and the width of the tumours xenografts respectively. Tumors were surgically excised at 33 weeks post injection (p.i.) and processed for histology. For spontaneous metastasis assay, mice were euthanized at the end-point of the experiment, and lungs were analyzed for mCherry expression.

For experimental metastasis assays, a group of 10 female nu/nu mice (Charles River) aged 7 weeks, were inoculated with MCF7 control, GSDMB-1 and GSDMB-2 cells (1×10^5^ in 0.1 ml sterile PBS) stably expressing the protein luciferase into the left ventricle of the heart by nonsurgical means. A successful intracardiac injection was indicated on day 0 by images showing systemic bioluminescence distributed throughout the animal. Only mice with evidence of a satisfactory injection continued in the experiment. Live animal bioluminescence optical imaging was performed as described [16] using the IVIS Spectrum system or the IVISR Lumina II system (Caliper, Xenogen). Measurements were taken weekly starting 1 week after injection. At the end-point of the experiments, mice were euthanized 5 min later of *in vivo* bioluminiscent measure, and organs were analyzed for luciferase expression. Data were quantified with the Living Imaging software 4.2 (Xenogen Corporation). Mice were housed and maintained under specific pathogen-free conditions and used in accordance with institutional guidelines and approved by the Committee for Animal Care from the Universidad Autonoma de Madrid (UAM).

To validate GSDMB expression in primary tumors an immunohistochemical staining was performed using LSAB method (Dako) with a heat-induced antigen retrieval step. Sections were immersed in boiling 10 mM sodium citrate at pH 6.5 for 2 min in a pressure cooker and rat anti-HA antibody (Roche) was used. The primary antibody was omitted in the negative controls. GSDMB staining was defined as positive for those samples with more than 5% of GSDMB-expressing tumour cells.

### Survival analysis in breast cancer gene expression datasets

To study the clinical value of GSDM genes in breast cancer, we retrieved from the public database ROCK (http://rock.icr.ac.uk) the gene expression data and its associated clinical information of the Cancer Genome Atlas Network (TCGA) study [17]. The normalized expression of GSDM gene probes (GSDMA: A_23_P152605; GSDMB: A_23_P66451; GSDMC: A_23_P60116; and GSDMD: A_24_P363738) was available for 534 breast cancer samples. For each of these genes, tumors were categorized as having "high expression" if the gene expression value was within the third percentile (top 25% expression from all the samples); otherwise they were categorized as "low". Overall-survival Kaplan Meier curves were generated and differences in survival were assessed by Log-rank test (p<0.05 considered as statistically significant) using GraphPad PRISM 4.0.

Additionally, we performed a combined analysis on six expression microarray datasets of breast cancer samples with clinical data [18]-[23]. Data from these studies were extracted from the wider microarray compilation by Ur-Rehman et al provided in the ROCK database. All these studies were carried out on the HG-U133A platform manufactured by Affymetrix, and data was subject to quality control and RMA normalization, as detailed in ROCK (http://www.rock.icr.ac.uk/search/viewSampleDetails2.jsp?projectid=196&manufacturer=Affymetrix). Next, the normalized expression values of *GSDMB* probe (219233_s_at) were median centered, and categorized as "high" when they were within the third percentile (top 25% expression). For survival analysis, tumors with disease free survival data (n = 1094, median follow-up time = 85 months) and distant metastasis free survival (n = 902, median follow-up time = 87 months) were selected and analyzed by Log-rank test.

### Statistical analysis

Data were tested for normality and paired sets of data were compared using paired Student's t-test (two tailed). Comparisons between multiple treatment groups from the same experiment were made using one-way ANOVA. When significant differences were found between groups, Bonferroni posttest was used to test significance. In all cases values of p<0.05 were considered statistically significant. These analyses were carried out using the GraphPad PRISM 6.0 software.

## Results

### *GSDMB* over-expression is associated to poor prognosis in breast carcinomas

**Figure 1:**
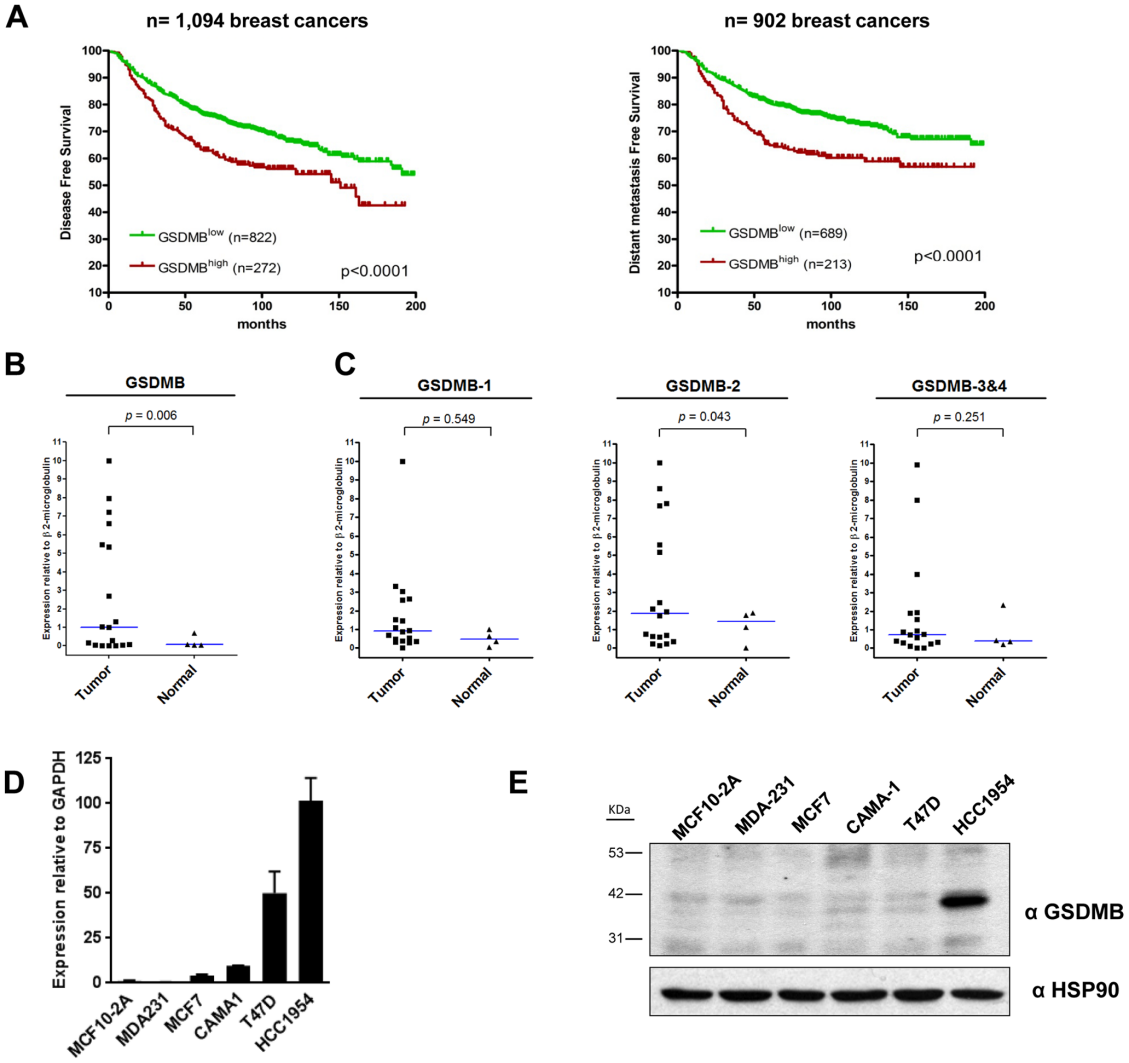
Expression of GSDMB in breast carcinoma and breast cancer cell lines. (A) Kaplan-Meier analysis of disease free survival (left) and distant metastasis (right) in relation to *GSDMB* expression in breast carcinoma data sets [18]–[23]. Tumor samples were classified as GSDMB^high^ (carcinomas with the top 25% highest expression levels of *GSDMB* gene) and GSDMB^low^ (rest of the samples). Differences in survival between the groups were assessed by log-rank test (*p*<0.0001). (B) Analysis of *GSDMB* expression in breast tumors samples (n = 18) and normal mammary tissue (n = 4) by qRT-PCR. Median of GSDMB expression relative to β2 microglobulin as housekeeping gene is shown in blue (C) Analysis of the expression of *GSDMB* splicing variants (GSDMB-1, -2, and 3&4) by qRT-PCR in the same breast tumors and normal tissue analyzed in B. Medians of expression relative to β2 microglobulin as housekeeping gene are shown in blue. p-values shown were calculated by Wilcoxon rank sum test. (D) Quantitative RT-PCR analyses of *GSDMB* in the indicated breast cell lines. Expression levels are relative to GAPDH. Bars represent the mean value ± s.d. of two independent experiments. (E) Analysis of GSDMB expression by western blot in the indicated cell lines. α-HSP90 was used as a loading control.

To evaluate the potential relevance of *Gasdermin* genes in breast cancer, we first tested whether their levels of expression were associated with breast cancer prognosis. We performed survival analyses based on their expression in a large series of breast carcinomas using publicly available gene expression dataset (Figure S2). The analysis in the TCGA dataset, comprising 534 breast cancers [17] evidenced that patients with tumors expressing high levels of GSDMB showed a significant reduction in overall survival (*p* = 0.018), while we could not find any association with prognosis for the other members of the family (Figure S1). To explore further the clinical relevance of GSDMB expression in breast cancer, we realized an *in silico* study combining six breast cancer profiling datasets performed on the HG-U133A Affymetrix microarray platform [18]–[23]. This analysis demonstrated that high levels of *GSDMB* expression were significantly associated with poor disease outcome, in both disease free survival (DFS, p<0.0001) and distant metastasis free survival (DMFS, p<0.0001) (Figure 1A).

Next, we evaluated *GSDMB* expression and its splicing variants in breast cancer tumors as well as normal mammary tissue by qRT-PCR (Figure 1B, C) *GSDMB* gene expression was significantly increased in breast carcinomas compared to normal samples (p = 0.006) (Figure 1B). The comparative analysis of GSDMB isoforms demonstrated that *GSDMB-2* expression was significantly increased in breast cancer tumors relative to normal mammary tissue (p = 0.043), while the rest of GSDMB isoforms did not show statistically significant differences (Figure 1C).

These data indicate that high levels of *GSDMB* expression are associated with poor disease outcome (both disease free survival and distant metastasis free survival) in breast carcinomas. Among GSDMB isoforms, *GSDMB-2* seems to be the most expressed isoform in breast cancer tumors.

Additionally, we examined *GSDMB* expression in a panel of breast cancer cell lines (MDA-231, MCF7, CAMA-1,T47D, HCC1954) as well as non-tumorogenic cell line (MCF10-2A) (Figure 1D-E). High-medium GSDMB mRNA expression levels were detected in T47D and HCC1954 cell lines; by contrast MDA-MB-231 and MCF7 showed low-levels of GSDMB. Moreover, non-expression of GSDMB was detected in non-tumorogenic cell line MCF10-2A (Figure 1D). Importantly, strong protein expression was detected only in HCC1954 cells (Figure 1E), which exhibit the highest levels of GSDMB mRNA.

### Morphological and phenotypic changes after GSDMB-1 and -2 over-expression in the MCF7 breast cancer cell line

To understand the implication of GSDMB overexpression in breast cancer pathogenesis we first analyzed the phenotypic effect of GSDMB over-expression on the MCF7 breast cancer cell line. This cell line presents low mRNA expression level of all the GSDMB isoforms (Figure S3 A) and no detectable GSDMB protein expression [8] compared with HCC1954 cells (Figure 1E). For this study we used GSDMB-2, the isoform most over-expressed in breast cancer samples, and GSDMB-1 which was not found significantly upregulated in the analyzed breast carcinoma samples (Figure 1C).

**Figure 2:**
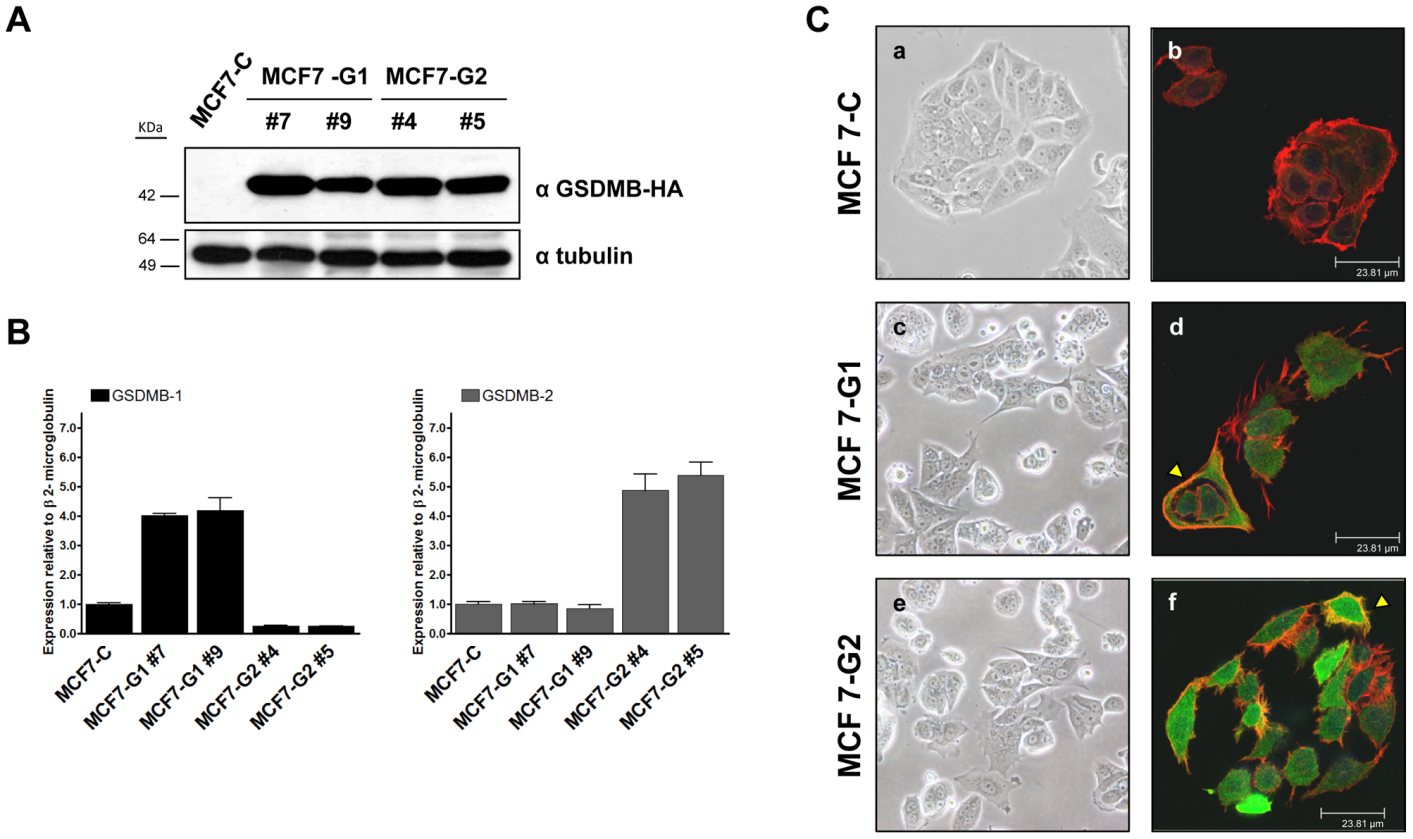
Stable expression of GSDMB-1 and -2 in MCF7 breast carcinoma cell line induces phenotypic changes. (A) Analysis of *GSDMB-HA* expression by western blot in control (MCF7-C), GSDMB1-HA (MCF7-G1) and GSDMB-2-HA (MCF7-G2) cells. α-tubulin was used as a loading control. Two independent clones of GSDMB-1 (#7, #9) and GSDMB-2 (#4, #5) are shown. (B) Quantitative RT-PCR analysis of GSDMB-1 and -2 in MCF7-C and GSDMB-1/2-transfected cells. Expression shown is relative to β2microglobulin that was used as housekeeping gene. (C) Phase-contrast images of MCF7-C, MCF7-G1 and MCF7-G2 cells, x20 magnification (panels a, b, c respectively). Confocal immunofluorescence staining shows the subcellular colocalization of GSDMB (GSDMB-HA in green) and Fibrilar-actin (in red) in MCF7-C, MCF7-G1 and MCF7-G2 cells (panels d, e, f). Bar  = 23.81 μm.

For this purpose, we generated MCF7 stable transfectants by over-expressing *GSDMB-1* (MCF7-G1) and *GSDMB-2* (MCF7-G2) transcripts tagged with hemagglutinin epitope (HA) (Figure 2). The overexpression of both isoforms was demonstrated at protein (Figure 1A) and mRNA level (Figure 2B). Importantly, we confirmed that exogenous expression of GSDMB-1 and -2 transfected cells was similar to the endogenous GSDMB expression levels observed in HCC1954 cells by western blot (Figure S3 B).

Interestingly, we observed that *GSDMB-1*/-*2* over-expression induced several morphological changes as demonstrated by the increase of membrane projections (Figure 2C, panels c, d, e, f) compared to control cells (Figure 2C, panels a, b). These results suggest that *GSDMB-1* and -*2* over-expression leads to a re-organization of membrane projections typical of a motile/invasive cell phenotype.

In addition, immunofluorescence staining and confocal microscopy analysis showed that both isoforms were predominantly present in the cytosol (Figure 2C). This is consistent with the cytosolic localization of endogenous GSDMB in HCC1954 cell line as shown by cell fractionation (Figure S3 C).

### *GSDMB* over-expression promotes cancer cell migration and invasion

**Figure 3:**
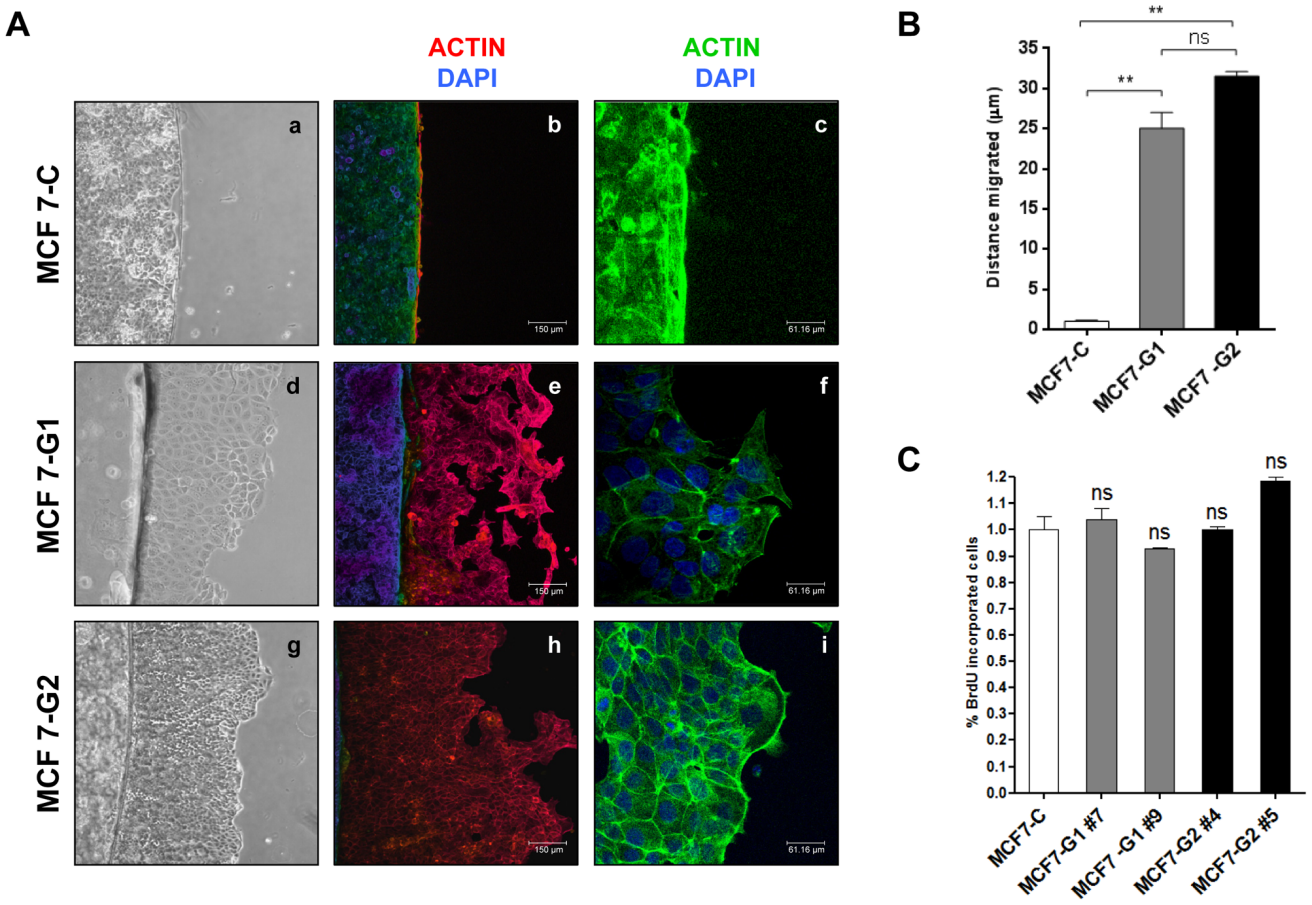
Over-expression of *GSDMB-1/-2* increases cell motility. (A) Analysis of cell motility in MCF7-C, MCF7-G1 and MCF7-G2 cells by barrier assay. Images were taken at five days post-seeding. Panels a, d and g show phase-contrast images. Panels b-c, e-f, h-i show F-actin staining by confocal immunofluorescence in the indicated cells. Cell nuclei were counterstained with DAPI (blue). Panels c, f and i show a magnification of the morphology of migrating cells at the invasive front for each cell line. Bars  = 150 μm middle panels, 61.16 μm right panels. (B) Quantification of the migrated distance from the edge of the coverslip (in μm) in barrier assays. Bars represent the mean value ± s.d. relative to control cells (MCF7-C) by one-way ANOVA test **0.001<*p*<0.005. N = 3 independent experiments. (C) Proliferation assay by BrdU incorporation in MCF7-C control cells and MCF7-G1 (#7,#9) and MCF7-G2 (#4, #5) transfectant clones. Bars represent the mean value ± s.d. by one-way ANOVA test; ns, non-significant. N = 3 independent experiments.

To characterize further the effect of GSDMB on cell motility, we performed barrier migration assays (Figure 3). We observed that cells over-expressing GSDMB-1/-2 exhibited an increase in cell migration, demonstrated by their ability to migrate out of the glass cover slip barrier, and by the formation of very dynamic cell protrusions characteristic of migrating cells (Figure 3A, panels d, e, g, h). In contrast, control cells were unable to move outside of glass cover-slip barrier (Figure 3A, panels a, b). Although, the cell migration dynamics of MCF7-G1 and MCF7-G2 were similar, MCF7-G2 cells showed a more active migratory behavior than MCF7-G1, 32 and 25 fold respectively (Figure 3B). Furthermore, cells transfected with the isoform 1 showed a less cohesive phenotype, evidenced by the formation of intercellular spaces between cells (Figure 3A, panel e, f); while MCF7-G2 cells appeared to have a more cohesive phenotype (Figure 3A, panels h, i). Immunofluorescence analysis showed that the actin filament network is distributed cortically in MCF7-G2 cells, and the invasion front had active membrane projections, presumably to generate the tracking force necessary for cell migration (Figure 3A, panel i).

To rule out that the increased cell migration was due to higher cell proliferation, we analyzed the proliferation of these cell lines by 5-bromo-2'-deoxyuridine (BrdU) incorporation into DNA. We did not find significant differences in the proliferation rate of the different cell types (Figure 3C). Therefore, cell growth does not seem to be contributing to the differences found in cell migration.

**Figure 4:**
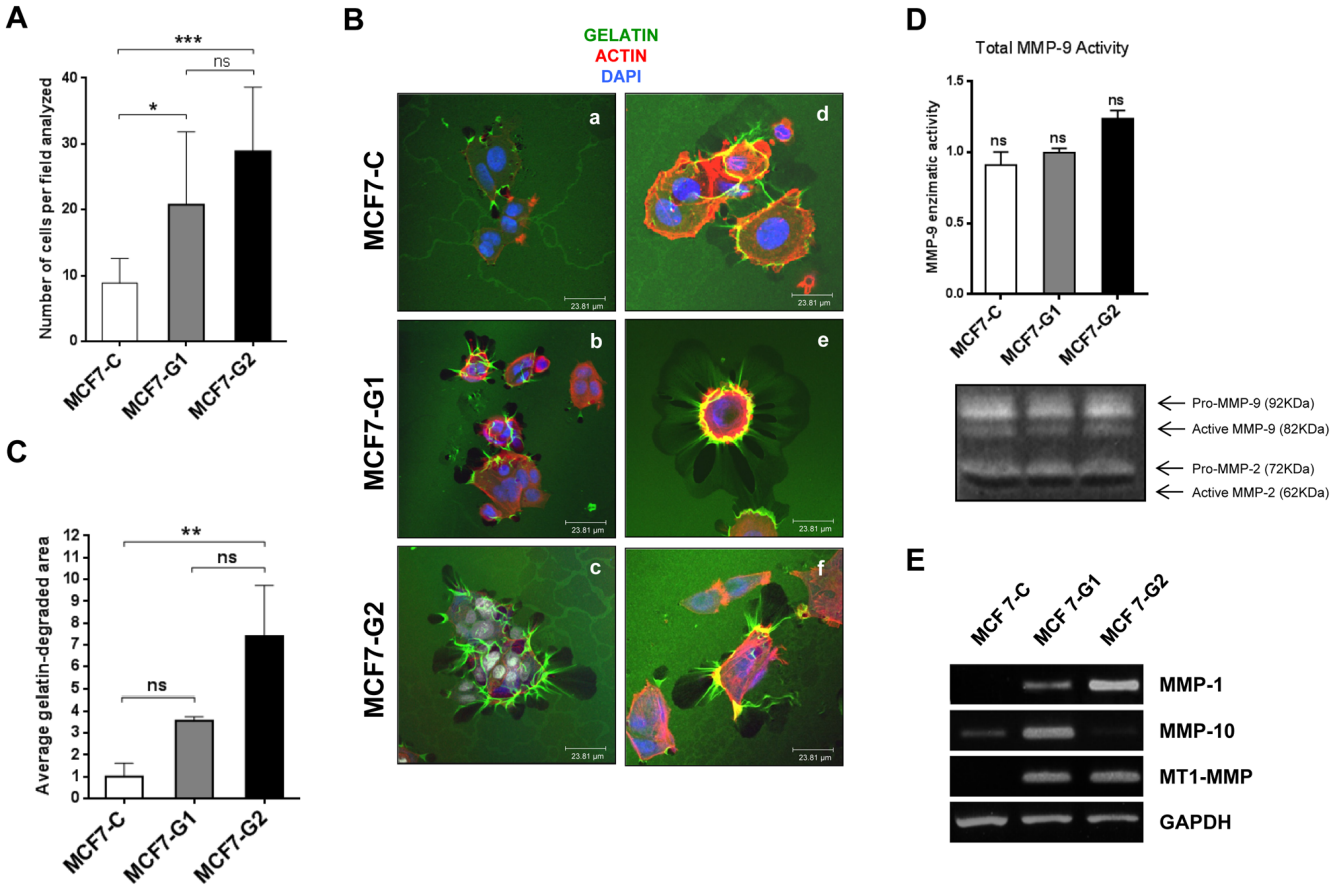
*GSDMB-1/-2* over-expression increases cell invasion and gelatin degradation. (A) Matrigel invasion assays of MCF7-C and MCF7-G1 and MCF7-G2 cells; bars represent the mean value ± s.d. by one-way ANOVA test **0.001<*p*<0.005;***p<0.001; ns, non-significant. N = 3 independent experiments by triplicate. (B) Representative confocal immunofluorescence images of fluorescent gelatin degradation assay in MCF7-C and MCF7-G1 and MCF7-G2 cells after 8 hours. F-actin and nuclei were stained with 546-phalloidin (red) and DAPI (blue) respectively. Degraded areas are visualized in black. Bar  = 23.81 μm. (C) Quantification of the gelatin-degraded area in GSDMB-1/-2 cells. Bars represent the mean value ± s.d. relative to control cells (MCF7-C) by one-way ANOVA test **0.001<*p*<0.005; ns, non-significant. N = 3 independent experiments. (D) Zymogram of MMP-9 and MMP-2 activity in the conditioned media from MCF7-C, MCF7-G1 and MCF7-G2 cells analyzed 24 hours after cell seeding. Graph shows the quantification of the relative band intensities calculated by densitometry analysis as described in the Materials and methods section. (E) Semi-quantitative RT-PCR analysis of the expression of MMP1, MMP10 and MT1-MMP in control MCF7-C cells and MCF7-G1 and MCF7-G2. GAPDH was used as housekeeping gene.

To determine if *GSDMB* over-expression increases cancer cell invasion we performed matrigel-coated Boyden chamber invasion assays. We found that *GSDMB-1*/*-2* over-expression dramatically increased the invasiveness of these cell lines compared to control cells (∼2.7 and ∼3 fold, respectively) (Figure 4A).

To confirm that GSDMB promotes migratory and invasive capacities in breast cancer cells, we analyzed the behavior of HCC1954 after stable knockdown of GSDMB. Using two shRNAs we efficiently knocked down GSDMB protein, by targeting various isoforms at the same time (Figure S4 A). Importantly, GSDMB repression resulted in a marked decrease of the migratory ability (Figure S4 B) as well as the invasion capacity of HCC1954 cells (Figure S4B), while the proliferation was not significantly affected (Figure S4 A).

To further investigate the increased in the invasive behavior of MCF7-GSDMB-1/-2 cells we evaluated their matrix degradation capacity using gelatin degradation assays on FITC-labeled gelatin (Figure 4B). We analyzed the local proteolytic activity by the appearance of dark areas lacking fluorescence in the bright fluorescent matrix. As shown in Figure 4B (panels a, d) degraded areas were scarcely detected in the control cells whereas in the GSDMB over-expressing transfected cells a significantly increase of these areas of degradation were observed (Figure 4B, panels b, c, e, f). The staining with phalloidin revealed also that F-actin rich cores extended from the basal surface of the cell into the cytosol and often co-localized with areas of matrix degradation in MCF7-G1 and MCF7-G2 cells (Figure 4B, panels e, f). The quantification of the degraded area revealed that although all cell lines show gelatinase activity, in MCF7-G2 cell line the degradation of substrate was significantly increased (∼7 fold), compared to MCF7-G1 (∼3.5 fold) as well as control cells as expected (Figure 4C). These data indicate that GSDMB-2 over-expression significantly increased proteolytic gelatinase activity.

It is well known that the extracellular matrix-degrading ability is largely dependent on matrix metalloproteinases (MMPs), including MT1-MMP, MMP-2, and MMP-9 [25], [26]. Based in our gelatin-based assays, we analyzed the secretion levels of different members of the MMP family in zymogram assays, but we could only detect the active form of MMP-9 (Figure 4D). Although both transfectants showed an increase in the activity of MMP-9, the quantification of observed differences was not significant (data not shown). Then, we studied the expression of a panel of 10 different proteases by RT-PCR. As shown in Figure 4E, we found an increased expression of MT1-MMP, MMP-1 and MMP-10 in GSDMB expressing clones compared to control cells. Interestingly, we found that there is a specific pattern of MMPs depending on the isoform: while MT1-MMP was overexpressed in both isoforms relative to control cells, MMP-1 was upregulated in GSDMB-2 cells, and MMP10 was increased only in GSDMB-1 cells (Figure 4E).

Collectively, our results suggest that GSDMB increases cell migration and invasion possibly by up-regulating the secretion of MMPs in an isoform-specific pattern.

### GSDMB promotes Rho GTPases activation

**Figure 5:**
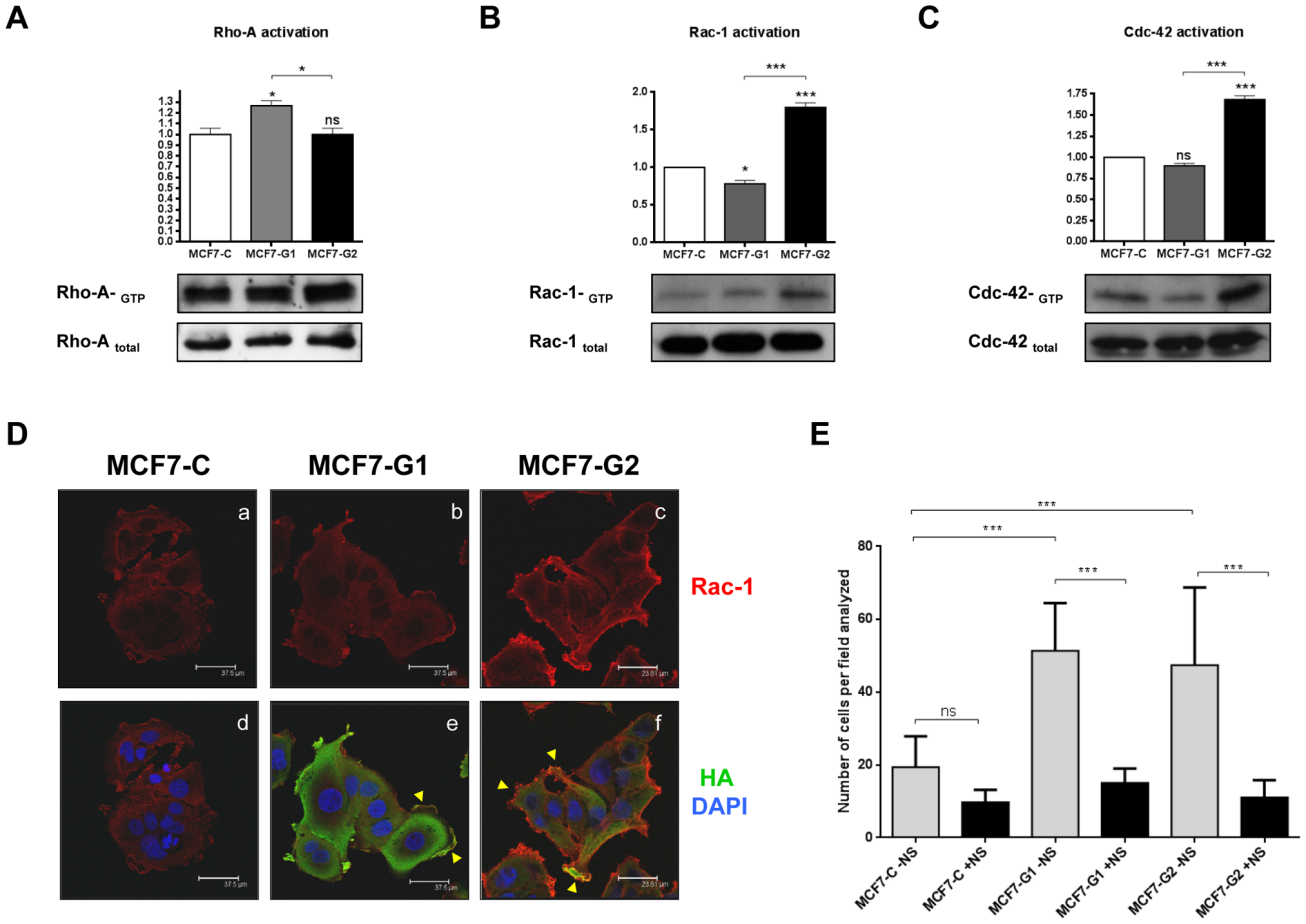
Rac-1 and Cdc-42 are activated in GSDMB-2 cells. Analysis of Rho A (A), Rac-1 (B) and Cdc-42 (C) activity by pull-down assays in MCF7-C, MCF7-G1 and MCF7-G2 cells. Densitometric analysis of Western blots analyzing each RhoGTPase activity is shown in the upper graphs. Bars represent the mean value ± s.d. relative to control cells (MCF7-C) by one-way ANOVA test *p<0.05;***p<0.001; ns, non-significant. (D) Confocal immunofluorescence analysis showing the localization of GSDMB-HA isoforms (green) and Rac-1 (red) in MCF7-C, MCF7-G1 and MCF7-G2 cells. Cell nuclei were counterstained with DAPI (blue). Bar: 37.5 μm. (E) Quantification of the effect of Rac-1 inhibition in cell migration. Cells were incubated in the absence of serum and treated with 100 μmol/L NSC23766 for 72 h. Untreated control cells were used for each condition. Bars represent the mean value ± s.d. relative to control cells (MCF7-C) by one-way ANOVA test *p<0.05; **0.001<*p*<0.005; *** *p*<0.001; ns, non-significant. N = 3 independent experiments.

To further investigate the function of GSDMB on cell migration, we analyzed the activation of several Rho-GTPases (Figure 5A-C). After pull down assays, we observed an increased activation of Rac-1 and Cdc-42 in GSDMB-2 expressing cells compared to control and to GSDMB-1 cells, as shown by higher levels of GTP-bound GTPases (Figure 5B, C). However, non-significant changes were observed in the Rho-A activation (Figure 5A). In addition, confocal immunofluorescence assays showed that Rac-1 staining was increased in GSDMB-2-expressing clones (Figure 5D, panels c, f) compared to control cells (Figure 5D, panels a, d), suggesting a role for this protein in the modification of motile structures. These findings are also in agreement with the increase in actin filaments observed by phalloidin staining in GSDMB-2 expressing cells (Figure 2C). To determine the involvement of Rac-1 in GSDMB-mediated migration, we performed migration assays in MCF7-GSDMB and control cells treated with NSC23766, a well-known Rac-1 inhibitor [27] (Figure 5E). Rac-1 inhibitor partially suppressed migration on MCF7-GSDMB-1 and GSDMB-2 cells, whereas this inhibitor did not significantly affect control cells (Figure 5E).

Together, these data suggest that the significant increases in the migration and invasion ability of GSDMB-2 cells could be related to the capacity of activating Rac-1 and Cdc-42 GTPases compared to GSDMB-1 and control cells.

### GSDMB-2 increases the tumorigenic and metastatic behavior in MCF7 cells

**Figure 6:**
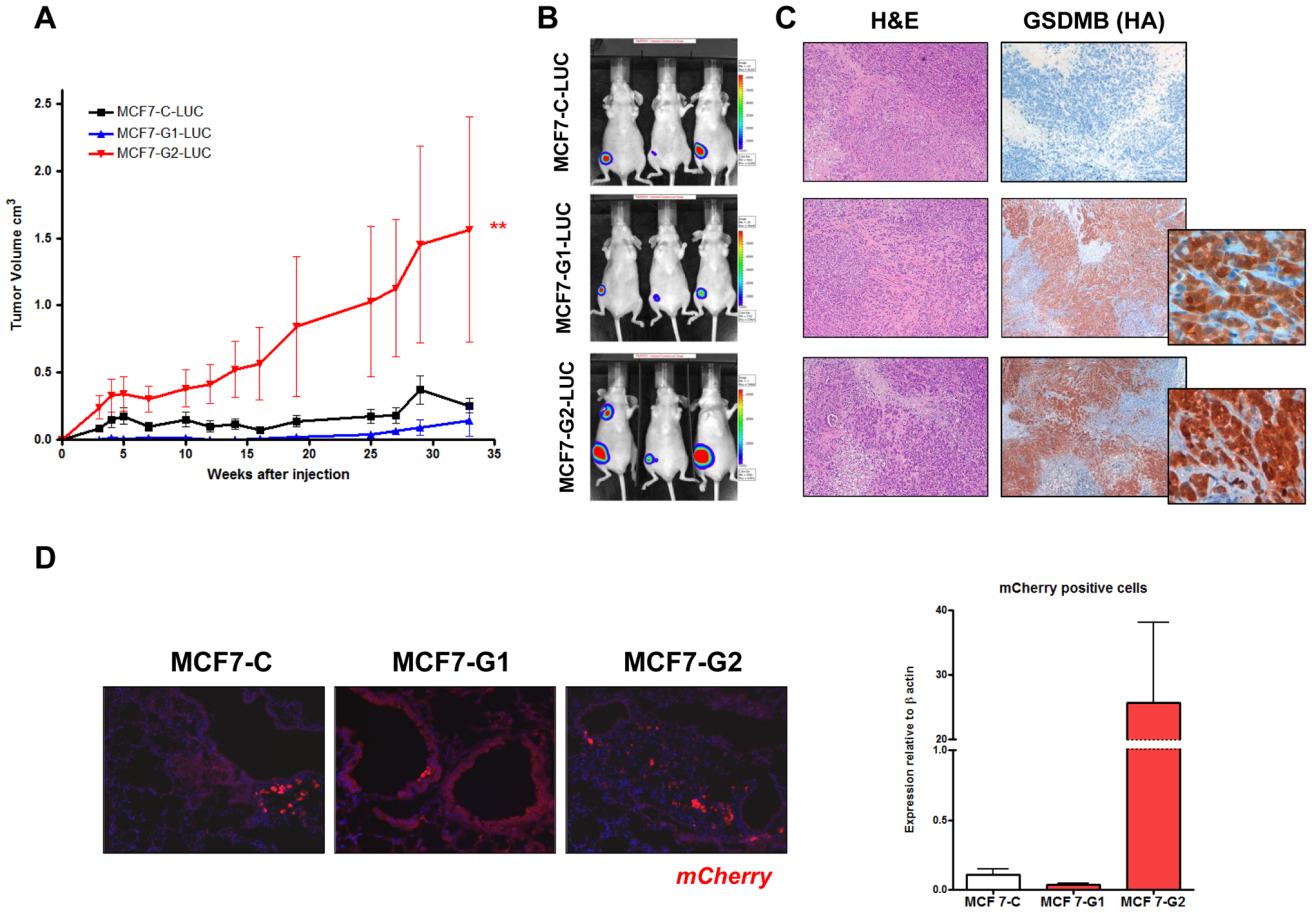
*GSDMB-2* over-expression increases the tumorogenic and the metastatic capacity of MCF7 cells. (A) Analysis of primary tumor growth after mammary fat pad injection of MCF7-C-LUC and MCF7-G1-LUC and MCF7-G2-LUC cells. Error bars represents the mean value ± s.d. **0.001<*p*<0.005 by two-way ANOVA. N = 5 mice per group (B) Representative bioluminescence images of mice injected with MCF7-C, MCF7-G1 and MCF7-G2-luciferase cells at week 33. The color scale represents the photon flux (photons per second) emitted. (C) Representative images of hematoxilin and eosin staining (left panels) and GSDMB-HA immunohistochemistry (right panels) in sections obtained from primary mammary tumors of the cells indicated (x20 magnification). Insets (right) are x60 magnification of the corresponding selected areas. (D) Analysis of metastases in mice injected with mCherry-cells (MCF7-C, MCF7-G1 and MCF7-G2). Representative images of mCherry protein expression (in red) within the lungs are shown on the left. Cell nuclei were counterstained with DAPI (blue), x40 magnification. Graph on the right shows the mCherry mRNA expression by qRT-PCR in the lungs of the indicated groups (right) relative to GAPDH.

In order to validate the biological involvement of GSDMB-1 and -2 in breast cancer progression, we evaluated their *in vivo* effect on tumor growth and metastasis using xenograft mouse models. Firstly, to track tumor cells and monitor tumor growth and metastasis, we stably over-expressed *mCherry* or luciferase proteins in MCF7 control, GSDMB-1 and GSDMB-2 transfectant cells. Analysis of tumor growth demonstrated that mice orthotopically injected with GSDMB-2 cells developed significantly bigger tumors (∼6 fold) at the end of the experiment (33 weeks) (Figure 6A, 6B), compared to control or GSDMB-1 cells. Surprisingly, mice injected with GSDMB-1 cells developed tumors smaller than the control group, although the difference was not significant (Figure 6A, 6B). After 33 weeks, tumors were excised and GSDMB expression was verified by immunohistochemistry with anti-HA antibody observing strong cytoplasmic localization of GSDMB-HA in the stable transfectant clones (Figure 6C, right panels and inset). Importantly, the analysis of spontaneous metastasis by immunofluorescence (Figure 6D, left panels) and qRT-PCR of mCherry expression (Figure 6D, right graph), demonstrated that GSDMB-2 overexpression increases metastatic burden of MCF7 cells by 24 fold compared to control and to GSDMB-1 expressing cells (Figure 6D).

**Figure 7:**
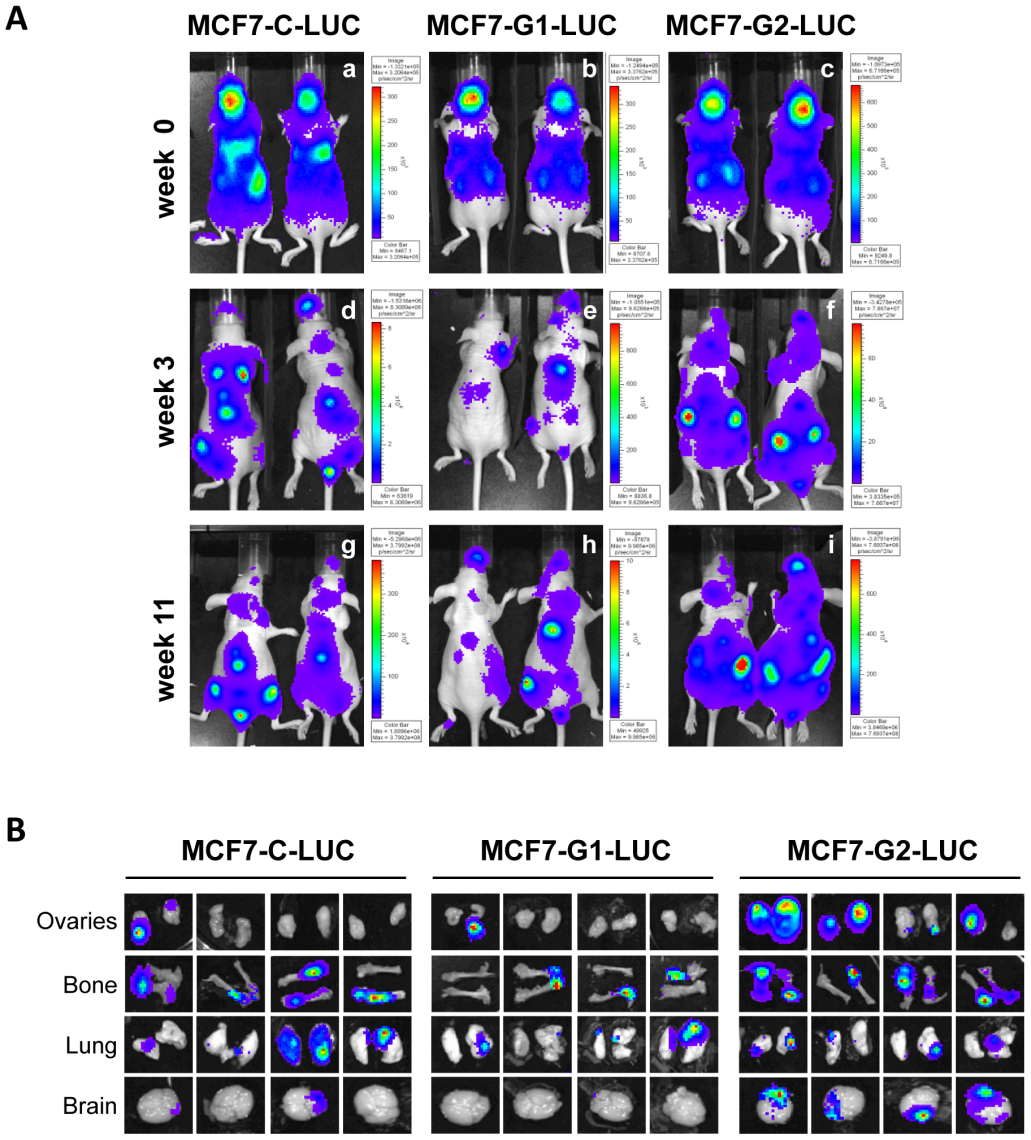
Analysis of cell homing and metastases of GSDMB transfectants by luciferase imaging. (A) Representative bioluminescence images of mice intracardially injected with luciferase-expressing MCF7-C (panels a, b, c), MCF7-G1 (panels d, e, f), and MCF7-G2 cells (panels g, h, j). Images were obtained at 0, 3 and 11 weeks after cell injection. The color scale represents the photon flux (photons per second) emitted from tumor cells. (B) Metastatic burden quantified by luciferin photon flux at 11 weeks after tumor injection (ovaries, bone, lungs and brain are shown) (C) Quantification of the percentage of organs with metastases. N = 5 mice per group. All experiments were performed in duplicate using 5 mice per experiment.

Furthermore, we evaluated the homing capacity and growth in metastatic organs using MCF7 control, GSDMB-1 and -2, stably expressing the protein luciferase and intracardially injected mice (Figure 7). After 3 weeks post-injection, all mice exhibited clear signs of distant metastatic lesions by luciferase imaging (Figure 7A, panels d, e, f). Mice were sacrificed after 11 weeks post-injection with large signs of metastatic dissemination by luciferase imaging at multiple sites, such as brain, lungs, abdomen and femurs, (Figure 7A, panels g, h, i). Representative *ex vivo* images of bones, lungs, ovaries and brain metastasis are shown in Figure 7B. We detected metastasis to lungs and bone-associated tissue in all mice with frequencies greater than 60-70% (Figure 7B, Table 1). Although all the cell lines showed a widespread pattern of spread, metastases to the brain and ovaries were substantially increased in GSDMB-2-injected mice, with frequencies greater than 90% in all the tissues analyzed (Figure 7B, Table 1). In contrast, mice injected with control cells showed metastases in only 20% of the ovaries and 60% of the brains analyzed. A similar frequency of metastases was observed in GSDMB-1 mice in both tissues (20% in ovaries and 20% in brain) (Figure 7B, Table 1).

**Table 1:** Metastasis incidence and frequency in multiple organs after intracardiac injection of MCF7 control (C) and transfected MCF7-G1 and MCF7-G2 cells.

	MCF7-C	MCF7-GSDMB-1	MCF7-GSDMB-2
**Ovaries**	2/10 (20%)	1/10 (10%)	9/10 (90%)
**Bone**	8/10 (80%)	6/10 (60%)	9/10 (90%)
**Lung**	9/10 (90%)	7/10 (70%)	8/10 (80%)
**Brain**	2/5 (40%)	1/5(20%)	5/5 (100%)

Number of organs affected in relation to all organs tested (in fraction and in percentage).

Taken together these data demonstrate that GSDMB-2 over-expression leads to an increased tumorogenic and metastatic behavior of MCF7 breast cancer cells, with enhanced metastatic cell homing and growth in ovaries and brain.

### GSDMB-2 is a novel client of Hsp90

**Figure 8:**
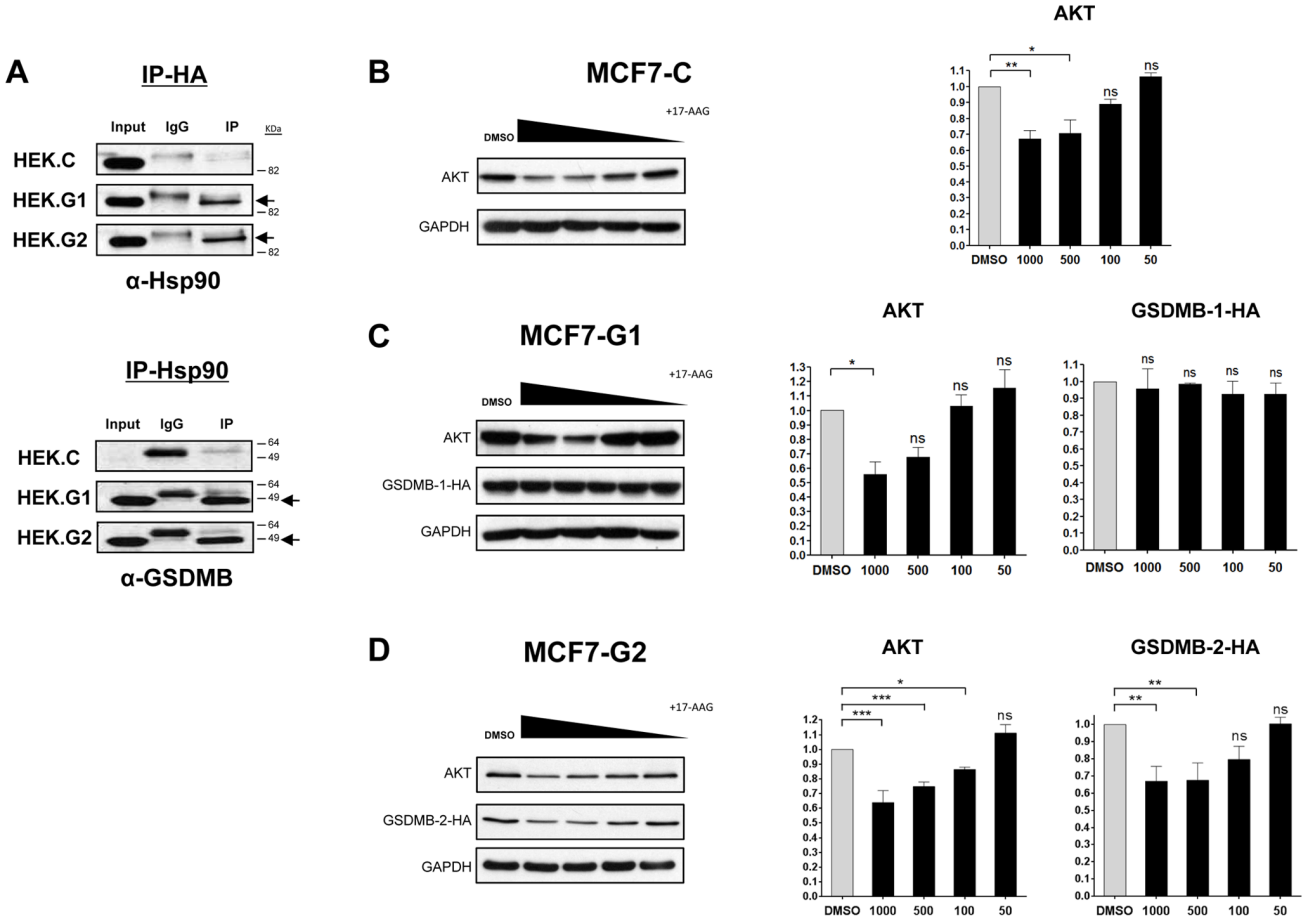
GSDMB-1 and GSDMB-2 interact with Hsp90 protein. (A) Immunoprecipitation assay of GSDMB-HA (left panel) and Hsp90 (right panel) in HEK293T cells transiently transfected with HA-tagged GSDMB-1 (HEK.G1) and GSDMB-2 (HEK.G2). After immunoprecipitation, specific interactions were detected using Hsp90 (α-Hsp90) and GSDMB (α-GSDMB) antibodies. (B, C, D) Analysis of protein levels by Western blot after treatment with the Hsp90 inhibitor 17 AAG (1,000, 500, 100, and 50 nmol/L) for 24 hours in MCF7-C cells (B), MCF7-G1 (C) and MCF7-G2 (D). GSDMB was detected using anti-HA antibodies. Akt detection was performed as positive control for Hsp90 inhibition. Densitometric analyses of the Wertern blots are shown in the right graphs. Bars represent the mean value ± s.d. relative to control (DMSO) by one-way ANOVA test followed by Bonferroni's posttest. *p<0.05; **0.001<*p*<0.005; *** *p*<0.001; ns, non-significant. N = 3 independent experiments.

To further characterize the functional role of GSDMB-1/-2 we identified their potential interacting partners by immunoprecipitation assays followed by mass spectrometry (MS; see Protocol S1 and S2 for detailed protocol). Using this approach, we identified Fatty acid synthase (FAS), and the Heat shock protein 90 β (Hsp90β) (Table 2), as new potential interacting partners of both GSDMB-1 and -2 isoforms. To validate the interaction, we performed analytical co-immunoprecipitation experiments using HEK293T cells transiently expressing GSDMB-1/-2-HA (Figure 8A). We confirmed that GSDMB-1 and GSDMB-2 interact with Hsp90 (Figure 8A) although we could not validate the interaction with FAS (data not shown). Next, to determine the functional consequences of the interaction between GSDMB-1 and -2 and Hsp90, we tested whether their protein levels were modified after treatment with the Hsp90 inhibitor 17-AAG (Figure 8C, 8D). As control, we used Akt (a well-known Hsp90-client affected by Hsp90 inhibition [28] and confirmed that its protein levels decreased in a 17-AAG concentration-dependent manner in all cell lines used (Figure 8A-D). Interestingly, we found that GSDMB-2 protein showed a significant reduction after Hsp90 inhibition, while the GSDMB-1 levels were almost stable (Figure 8C). These results demonstrate that GSDMB-1/-2 are new Hsp90-interacting proteins, however only GSDMB-2 seems to be a direct client of Hsp90, as its stability relies on Hsp90 activity.

**Table 2:** Peptides identified after immunoprecipitation followed by mass spectrometry assay in MCF7-G1 and MCF7-G2 cell lines.

Protein	Accession	Mass,	Score*	Peptides
**Name**	**number**	**KDa**		LSFFFDFR //
FAS	P49327	257	93	EQGVTFPSGDIQEQLIR //
(Fatty acid				LQVVDQPLPVR//
synthase)				HSQDLAFLSMLNDIAAVPATAMPFR //
				GYAVLGGERDNLEFFLAGIGR //
				LHLSGIDANPNALFPPVEFPAPR //
				MVVPGLDGAQIPR
**Hsp90**	P08238	90	289	IDIIPNPQER //
(Heat shock				TLTLVDTGIGMTK //
protein 90-β)				HFSVEGQLEFR //
				RAPFDLFENK //
				GVVDSEDLPLNISR //
				KHLEINPDHPIVETLR //
				HLEINPDHPIVETLR //

*Score is a parameter characterizing identification reliability of a certain protein. In general, at score value >60–70, identification may be considered as reliable.

## Discussion

The novel Gasdermin family of proteins (GSDMA-D) has been implicated in carcinogenesis and tumor progression [4]–[9] although their exact molecular and cellular function in these processes is not well known. Overall, previous literature proposed that *GSDMA* is considered a tumor suppressor gene according to its pro-apoptotic effect in cancer cells [2], [4], while *GSDMB* gene, which is up-regulated in some cancer types, might have a tumor-promoting role [5]–[9]. However, the potential involvement of *GSDM* genes in breast cancer has not been addressed before. Here we demonstrate for the first time that high levels of *GSDMB* gene, but not the other GSDM members, are associated to poor prognosis (in terms of disease-free and metastasis-free survival) in human breast carcinomas. Most importantly, we have uncovered that GSDMB promotes a pro-invasive and pro-metastatic role in breast cancer. Our work provide new clues about the role of GSDMB and its isoforms in cancer: a) there is a differential expression of GSDMB isoforms in breast cancer; b) GSDMB-1 and -2 trigger a pro-migratory and pro-invasive program in breast cancer cells; c) silencing of GSDMB reduces the migratory and invasive capacities in HCC1954 breast carcinoma cell line, d) GSDMB-2 plays an active role in tumor growth and metastasis; e) the identification of GSDMB-2 as a new interacting Hsp90 protein. All these new aspects of GSDMB will be discussed below.

Even though there are some previous reports focused on the expression of GSDMB, the function and the hypothetical relevance of GSDMB protein in breast cancer is still unknown. Therefore, we decided to investigate the role of this protein in breast cancer. The analysis of the expression of this molecule in human breast cancer suggests that GSDMB is over-expressed in breast carcinomas and it could be considered as a potential marker in these tumors. In fact, patients with increased expression of this molecule showed a reduced survival and increased metastatic disease. Our analysis of specific isoforms reveals that *GSDMB-2* is significantly upregulated in a cohort of breast cancer tumors as compared to other isoforms. These results suggest that GSDMB-2 could be responsible for the poor prognosis of tumors overexpressing GSDMB. Our data together with previous reports in gastric cancer [7], [8] indicate that GSDMB isoforms have differential expression levels within tumors and in normal tissues, and importantly, they may have distinct cellular localization and biological functions. Indeed, in MCF7 cells, both GSDMB isoforms are mainly detected in the cytoplasm, consistent with the cytoplasmic distribution of the endogenous GSDMB detected in HCC1954 cells and similar to the previously reported localization in hepatocellular carcinomas and gastric tumors [7]. Nonetheless, GSDMB-1 has also been described to show nuclear localization in cervix carcinomas [8].

Our analysis of *GSDMB* over-expression in the breast cancer cell tumor model MCF7 demonstrates that it promotes cell motility, invasion and metastasis whereas GSDMB silencing strongly reduced migration and invasion in HCC1954 breast cell line. Our results showed that GSDMB-1 and -2 may play a differential role in breast cancer. While the isoform 2 (GSDMB-2) seems to drive a tumorogenic and metastatic behavior in MCF7 cell line *in vitro* and *in vivo*, GSDMB-1 demonstrated a milder effect and could only be observed *in vitro*. In fact, it was reported that over-expression of *GSDMB-1* transcript did not promote tumorogenesis in nude mice using CHO cell line [8].

The differences in tumorogenicity between GSDMB-1 and 2 are surprising, as these isoforms differ only in exon 7, which is not present in isoform 2 (Figure S1). Although the functional relevance of exon 7 is unclear, based on our results, we hypothesize that the alternative use of this exon could enable the coordination of specific biological programs within the cell, given that the levels of exogenous expression in MCF7 cells of both isoforms are similar (at mRNA and protein levels). However, further data will be required to demonstrate this hypothesis.

The differences in the *in vivo* behavior between MCF7 cells expressing GSDMB isoforms 1 and 2 could be explained in part by the distinct activation of pro-migratory and pro-invasion molecules and pathways observed *in vitro*. Thereby, GSDMB-2 but not GSDMB-1 promotes activation of Rac-1 and Cdc-42. It is well established that Rac-1 and Cdc-42 proteins, which are frequently upregulated in human cancers, including breast cancer, contribute to tumor progression and metastasis [29]–[32]. Our data suggest that GSDMB-2 could promote increased cell migration *in vivo* through a Rac-1/Cdc-42- dependent mechanism, although we could not find a direct interaction with these proteins (data not shown). In addition to Rho GTPases, other molecules directly involved in breast cancer invasion and metastasis such as MMPs are differentially expressed after isoforms 1 and 2 over-expression. Activation of these molecules may contribute to the enhanced tumorogenicity and metastatic ability of GSDMB-2.

In addition to these intrinsic factors, GSDMB-2 may also activate extrinsic signals in the surrounding tissue that allow cancer cells to invade or colonize. It is worth noting, that while neither GSDMB-1 nor GSDMB-2 increases proliferation of MCF7 cells, GSDMB-2 promotes lung metastasis when injected in the mammary fat pad. As MCF7 cells are weakly metastasic in hormonally intact nude mice [33]-[35], in order to study the full metastatic potential of GSDMB-expressing cells, we performed intracardiac injection, which allows MCF7 cells to metastatize to multiple organs, including bones, lung, brain, and lymph nodes among others [36]–[37]. Using this system, GSDMB-2-expressing cells significantly increased the colonization of multiple organs such as brain, lungs, bones and ovaries, compared to GSDMB-1 and control cells. Taking together all this data it is conceivable that *GSDMB-2* over-expression confers an advantage required for metastatic dissemination.

The biological function of GSDMB is largely unknown, probably due to the lack of obvious functional domains. Moreover, to date the mechanisms controlling its stability and its potential interacting partners are unknown. In this sense our immunoprecipitation and mass spectrometry assays demonstrated that Hsp90 protein interacts with GSDMB-1 and 2. As Hsp90 and its related co-chaperones play a regulatory role in maintaining conformational maturation and structural integrity of a variety of cellular proteins [38]–[39], we analyzed if GSDMB could be a new client protein of this chaperone. Moreover, using *in silico* docking analysis we found that although both isoforms are hypothetically able to interact with Hsp90, the interaction between GSDMB-2 and HSP90 seems to be more energetically favorable (data not shown). More interestingly, we validated this study corroborating that, GSMDB-2 but not GSDMB-1 protein levels are regulated in an Hsp90-dependent manner. These data could suggest that the differential role of both isoforms could be attributed to the lack of this specific region, although this hypothesis remains to be demonstrated. Indeed, different studies have demonstrated that alternative splice isoforms of the very same kinase sometimes show striking differences in Hsp90 binding, suggesting that a distributed set of residues is required for robust Hsp90 association [40]. According to our results, Hsp90 inhibition could therefore be a novel mechanism to block GSDMB-2 and its tumorigenic potential.

In summary, we have shown that GSDMB up-regulation in breast cancer associates to poor prognosis and increased metastasis. The molecular mechanisms promoting GSDMB over-expression in breast carcinomas, though, remains to be elucidated. In gastric tissue, it has been suggested that cellular and viral origin LTR promoters could selectively control GSDMB expression in normal and cancer tissues, respectively [9], [41], [42], and that an Alu element, located in the upstream region of *GSDMB* gene, could be also responsible for *GSDMB* up-regulation [9]. Moreover, GSDMB gene amplification has been reported in a small subset of gastric carcinomas [5]. Whether similar mechanisms occur in breast cancer, will be the focus of future research. Independently of the mechanism of over-expression, we have demonstrated for the first time that GSDMB is functionally involved in breast cancer tumor aggressiveness and metastatic dissemination. Specific isoforms of this molecule seem to have a differential role in cancer and could be regulated by particular mechanisms. We propose that GSDMB could be considered as a new marker of invasiveness and metastasis in breast cancer, although additional studies will be required to fully understand the molecular mechanisms involved. Our work opens the way for future analysis of GSDMB proteins in breast cancer tumor progression and metastasis.

## Supporting Information

Figure S1 **Schematic representation of the exon–intron structure and the alternative splicing isoforms of the human GSDMB gene.** Schematic representation of the GSDMB gene exon–intron structure (top) and the splicing isoforms of the GSDMB gene as predicted by the NCBI database. An arrow marks the translational start site of GSDMB in exon 2 (E2). Black boxes represent coding exons, grey boxes show untranslated regions and introns are indicated by solid lines. The alternative processing of the exon 6 (E6) and 7 (E7) in the isoforms 1, 2 and 4 are represented by dotted lines.
(TIF)

Figure S2 **Survival analysis of Gasdermin genes in breast cancer. Association of the expression of Gasdermin genes (GSDMA-D) with overall survival in 534 patients with breast cancer.** Expression data was retrieved from The Cancer Genome Atlas Network study [17] and plotted as Kaplan Meier curves. For each gene, tumor samples were classified as high (carcinomas with the top 25% highest expression levels of *GSDM* genes) and low (the rest of tumors). Differences in survival between the groups were assessed by log-rank test.
(TIF)

Figure S3 **Comparison of endogenous versus exogenous GSDMB protein levels and subcellular distribution.** (A) Quantitative RT-PCR analyses of the expression of all the GSDMB isoforms in MCF10-2A, MCF7 and HCC1954 cells relative to GAPDH expression. (B) Western blot analyses to determine the relative amounts of endogenous protein levels of GSDMB (47 kDa) in HCC1954 cells versus the corresponding overexpressed variants (MCF7-G1 and MCF7-G2) and control cells (MCF7-C). GAPDH expression was used as housekeeping gene. (C) Subcellular fractionation and localization of endogenous GSDMB in HCC1954 cells. Equal amounts of whole cell (WCE: lane 1), cytoplasmic (CE: lane 2), membrane (ME: lane 3), nuclear (NE: lane 4), chromatin-bound (CB:lane 5) and cytoskeletal (PE: lane 6) extracts were loaded and incubated with anti-GSDMB antibody. The purity of these fractions was confirmed with antibodies against Calnexin (membrane), HSP90 (cytoplasmic), Snail2 (nuclear), HistoneH3 (chromatin-bound) and Cytokeratins (cytoskeletal).
(TIF)

Figure S4 ***GSDMB*** **knockdown in HCC1954 cells reduces the migration and invasion capacities.** (A) GSDMB expression in HCC1954 control cells (shControl) and in shGSDMB-derived cells (sh108, sh794). (B) Quantitative RT-PCR analyses of the expression of the different isoforms of *GSDMB* in control (shControl) and two different shGSDMB (sh108, sh794) generated in HCC1954 cells. (C) Cell proliferation was evaluated using alamarBlue assay in control (shControl) and shGSDMB (sh108, sh794) HCC1954 cells. Three independent experiments are represented as mean ± sd. Bars represent the mean value ± s.d. by one-way ANOVA test; ns, non-significant. (D) Quantification of transwell migration assay of shControl (shControl) and shGSDMB- HCC1954 derived cells (sh108 and sh794); Bars represent the mean value ± s.d. relative to control (shControl) by one-way ANOVA test *p<0.05; **0.001<*p*<0.005; *** *p*<0.001; ns, non-significant. N = 3 independent experiments. (E) Invasion assay on matrigel of control (shControl) and shGSDMB-HCC1954 derived cells (sh108 and sh794); Bars represent the mean value ± s.d. relative to control (shControl) by one-way ANOVA test *p<0.05; **0.001<*p*<0.005; *** *p*<0.001; ns, non-significant. N = 3 independent experiments.
(TIF)

Table S1 **Specific primers used for semiquantitative PCR.** Sequence of oligonucleotides, forward (F) and reverse (R), for sqRT-PCR. The amplicon size is indicated in pair bases (pb). sqRT-PCR conditions were optimized for each primer-pair. Amplification reactions consisted of following steps: 95°C for 5 min, 25–30 cycles at 95°C for 30 sec; optimized annealing temperatures for 30 sec and 72°C for 10 min.
(DOCX)

Table S2 **Primers used for qRT-PCR.** Reference of Taqman and SybGreen assays used in qRT-PCR. The manufactures is indicated in the table.
(DOCX)

Protocol S1 **Identification of proteins by Mass sectrometry (MS).**
(DOCX)

Protocol S2 **MALDI peptide mass fingerprinting and MS/MS analysis.**
(DOCX)
