# Supplementary figures and images for: Gasdermin-B Promotes Invasion and Metastasis in Breast Cancer Cells
Source: PLoS One. 2014 Mar 27;9(3):e90099. doi: 10.1371/journal.pone.0090099 (PMC3967990; doi:10.1371/journal.pone.0090099)

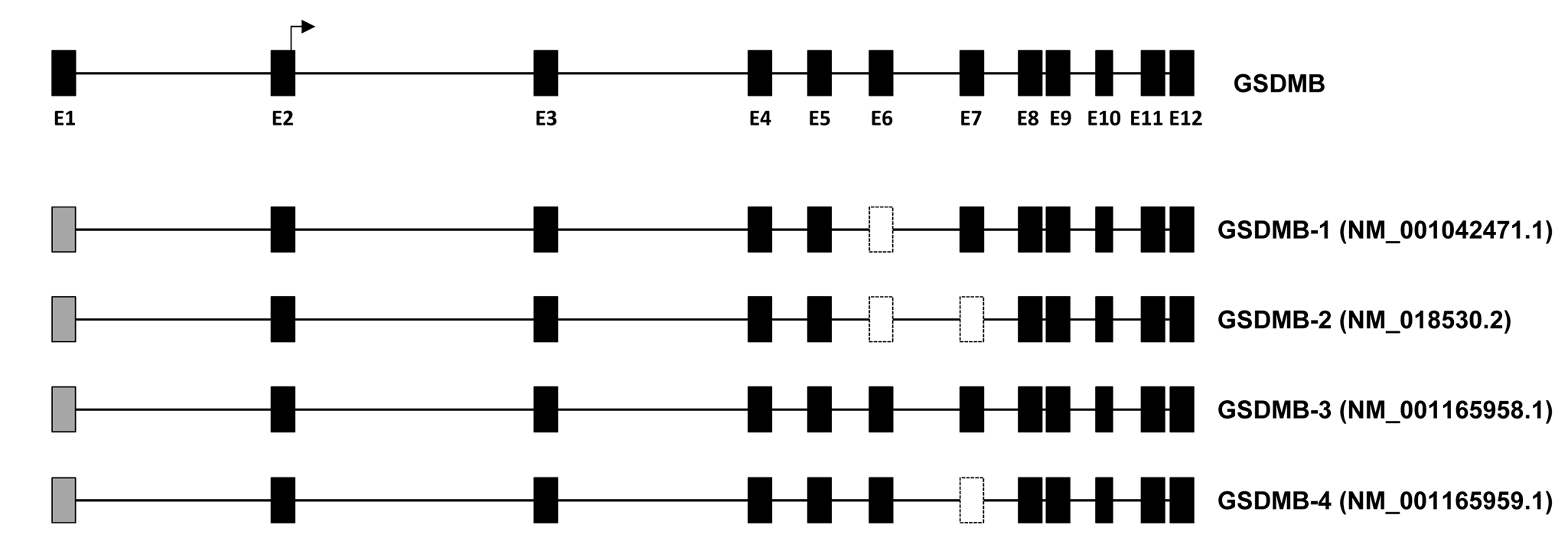

Supplement: Figure S1 — Schematic representation of the exon–intron structure and the alternative splicing isoforms of the human GSDMB gene. Schematic representation of the GSDMB gene exon–intron structure (top) and the splicing isoforms of the GSDMB gene as predicted by the NCBI database. An arrow marks the translational start site of GSDMB in exon 2 (E2). Black boxes represent coding exons, grey boxes show untranslated regions and introns are indicated by solid lines. The alternative processing of the exon 6 (E6) and 7 (E7) in the isoforms 1, 2 and 4 are represented by dotted lines. (TIF) [file pone.0090099.s001.tif]

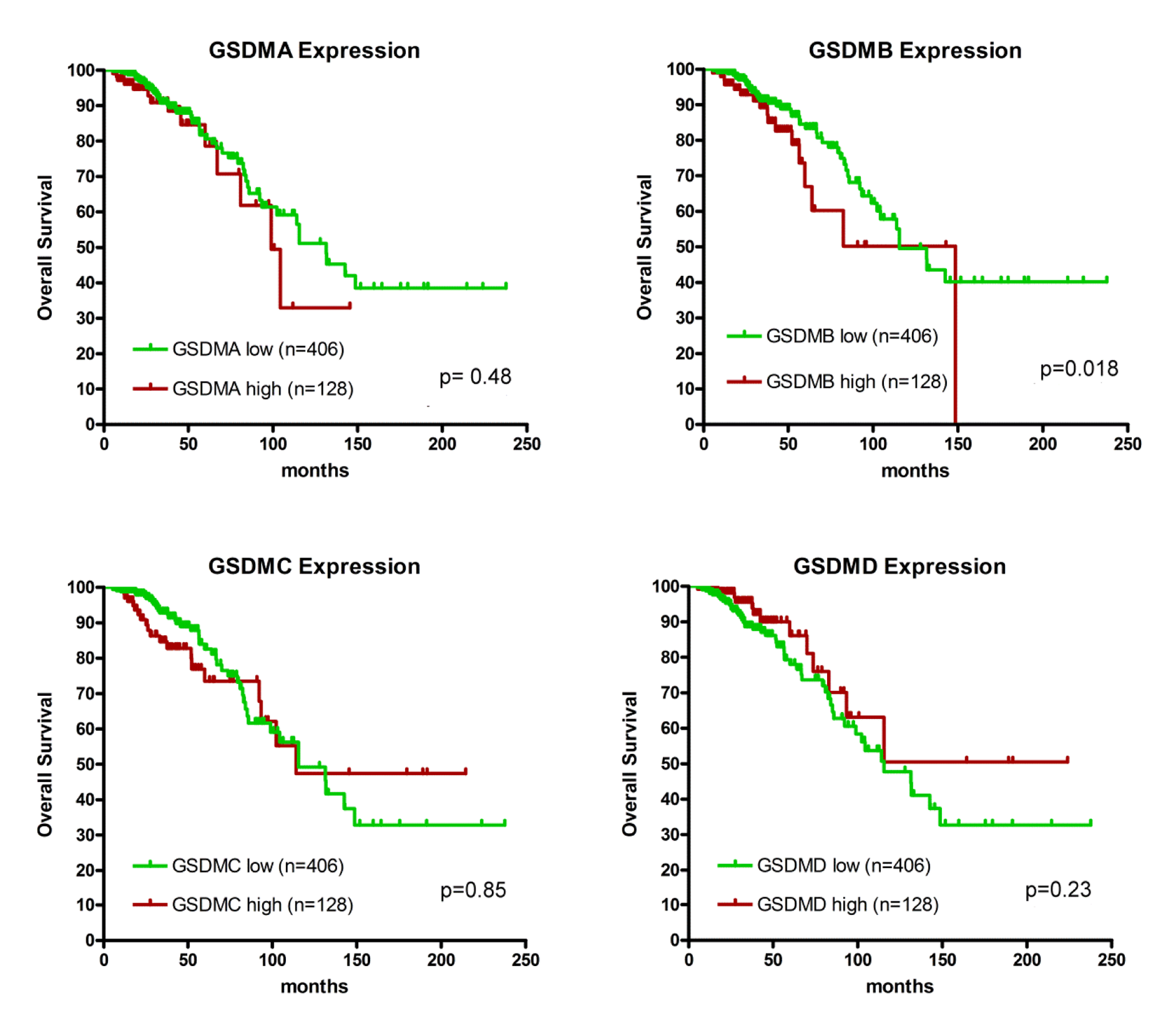

Supplement: Figure S2 — Survival analysis of Gasdermin genes in breast cancer. Association of the expression of Gasdermin genes (GSDMA-D) with overall survival in 534 patients with breast cancer. Expression data was retrieved from The Cancer Genome Atlas Network study [17] and plotted as Kaplan Meier curves. For each gene, tumor samples were classified as high (carcinomas with the top 25% highest expression levels of GSDM genes) and low (the rest of tumors). Differences in survival between the groups were assessed by log-rank test. (TIF) [file pone.0090099.s002.tif]

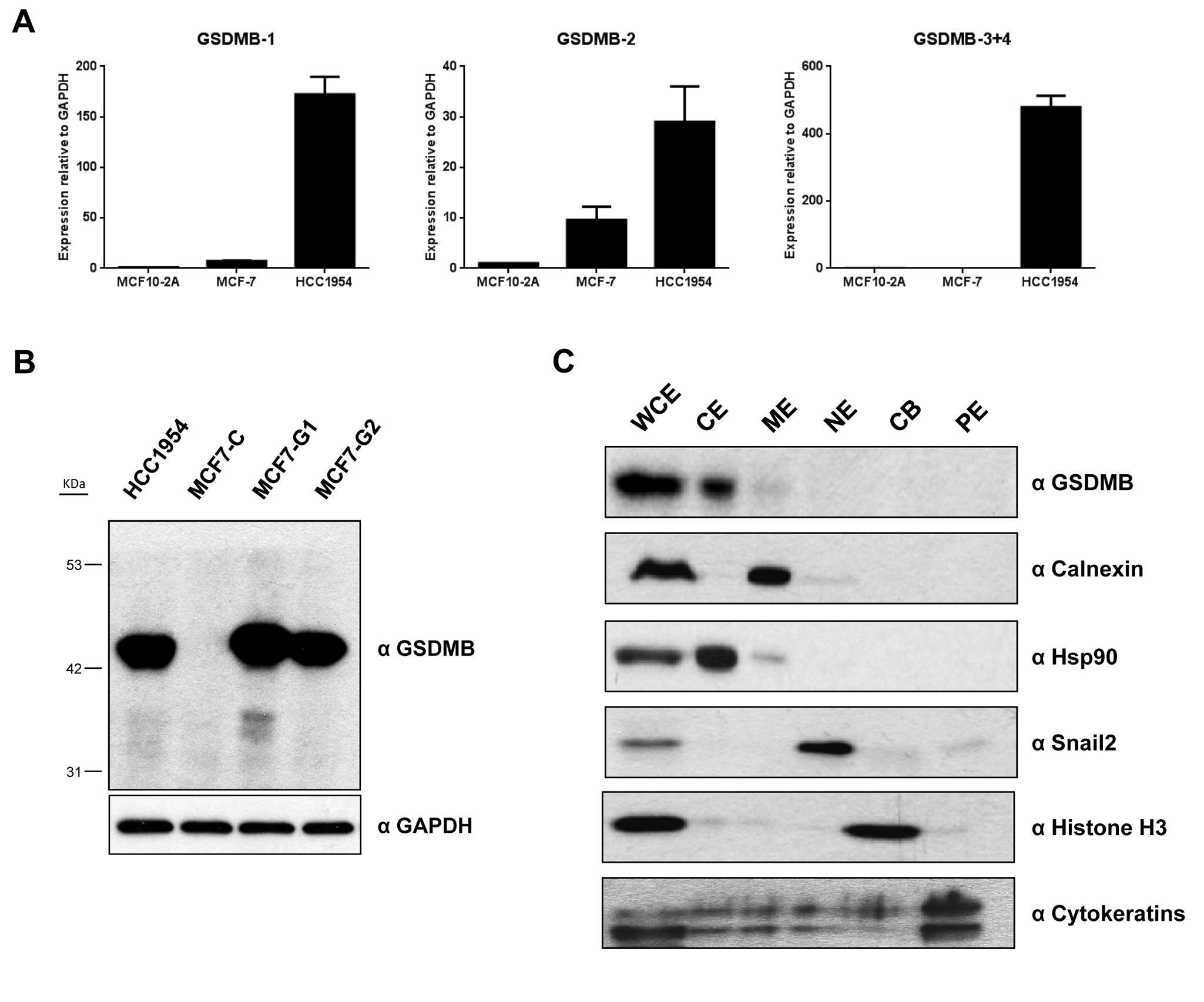

Supplement: Figure S3 — Comparison of endogenous versus exogenous GSDMB protein levels and subcellular distribution. (A) Quantitative RT-PCR analyses of the expression of all the GSDMB isoforms in MCF10-2A, MCF7 and HCC1954 cells relative to GAPDH expression. (B) Western blot analyses to determine the relative amounts of endogenous protein levels of GSDMB (47 kDa) in HCC1954 cells versus the corresponding overexpressed variants (MCF7-G1 and MCF7-G2) and control cells (MCF7-C). GAPDH expression was used as housekeeping gene. (C) Subcellular fractionation and localization of endogenous GSDMB in HCC1954 cells. Equal amounts of whole cell (WCE: lane 1), cytoplasmic (CE: lane 2), membrane (ME: lane 3), nuclear (NE: lane 4), chromatin-bound (CB:lane 5) and cytoskeletal (PE: lane 6) extracts were loaded and incubated with anti-GSDMB antibody. The purity of these fractions was confirmed with antibodies against Calnexin (membrane), HSP90 (cytoplasmic), Snail2 (nuclear), HistoneH3 (chromatin-bound) and Cytokeratins (cytoskeletal). (TIF) [file pone.0090099.s003.tif]

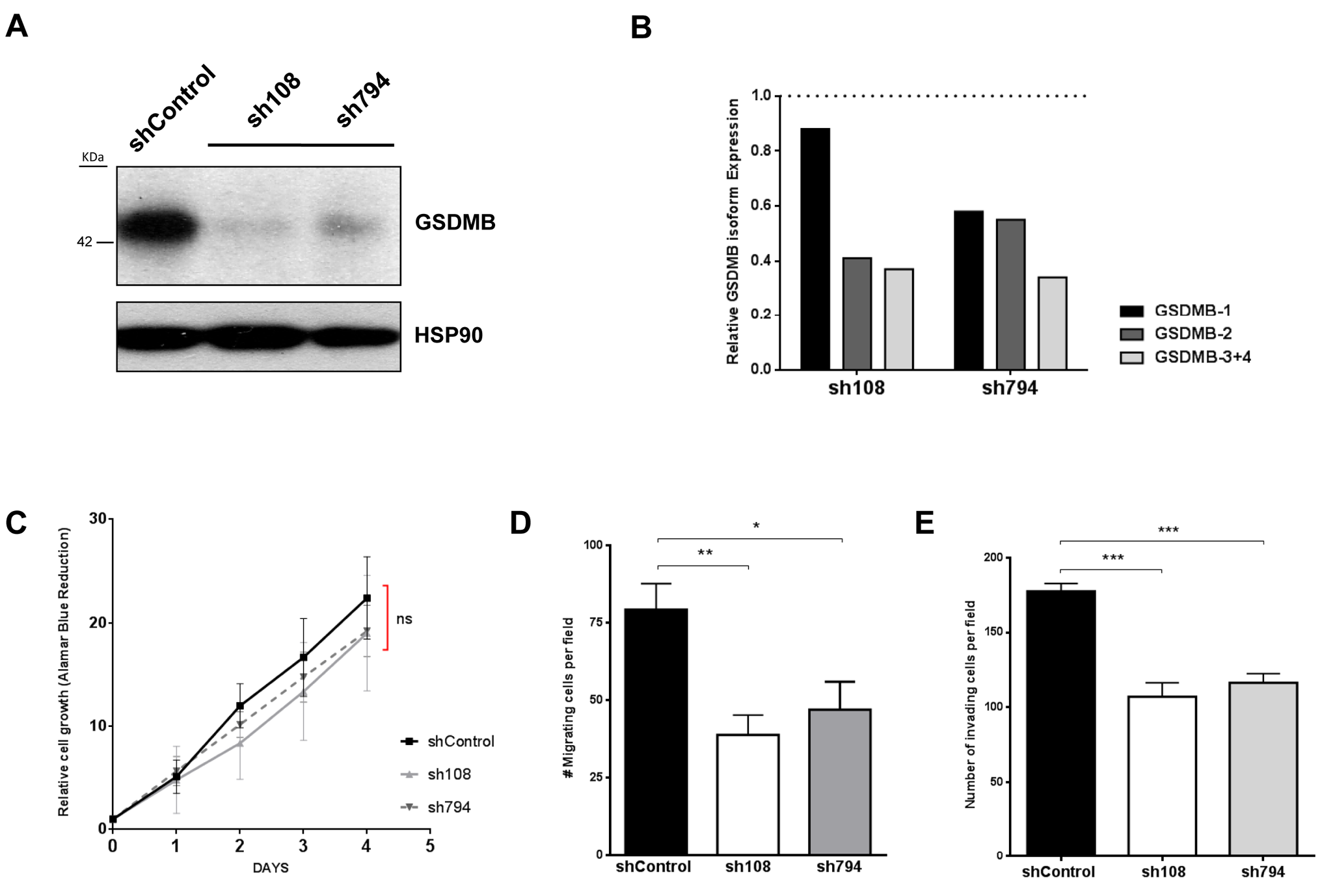

Supplement: Figure S4 — GSDMB knockdown in HCC1954 cells reduces the migration and invasion capacities. (A) GSDMB expression in HCC1954 control cells (shControl) and in shGSDMB-derived cells (sh108, sh794). (B) Quantitative RT-PCR analyses of the expression of the different isoforms of GSDMB in control (shControl) and two different shGSDMB (sh108, sh794) generated in HCC1954 cells. (C) Cell proliferation was evaluated using alamarBlue assay in control (shControl) and shGSDMB (sh108, sh794) HCC1954 cells. Three independent experiments are represented as mean ± sd. Bars represent the mean value ± s.d. by one-way ANOVA test; ns, non-significant. (D) Quantification of transwell migration assay of shControl (shControl) and shGSDMB- HCC1954 derived cells (sh108 and sh794); Bars represent the mean value ± s.d. relative to control (shControl) by one-way ANOVA test *p<0.05; **0.001<p<0.005; *** p<0.001; ns, non-significant. N = 3 independent experiments. (E) Invasion assay on matrigel of control (shControl) and shGSDMB-HCC1954 derived cells (sh108 and sh794); Bars represent the mean value ± s.d. relative to control (shControl) by one-way ANOVA test *p<0.05; **0.001<p<0.005; *** p<0.001; ns, non-significant. N = 3 independent experiments. (TIF) [file pone.0090099.s004.tif]
