# Supplementary material for: Gasdermin-B Promotes Invasion and Metastasis in Breast Cancer Cells
Source: PLoS One. 2014 Mar 27;9(3):e90099. doi: 10.1371/journal.pone.0090099 (PMC3967990; doi:10.1371/journal.pone.0090099)
Supplement: Table S1 — Specific primers used for semiquantitative PCR. Sequence of oligonucleotides, forward (F) and reverse (R), for sqRT-PCR. The amplicon size is indicated in pair bases (pb). sqRT-PCR conditions were optimized for each primer-pair. Amplification reactions consisted of following steps: 95°C for 5 min, 25–30 cycles at 95°C for 30 sec; optimized annealing temperatures for 30 sec and 72°C for 10 min. (DOCX) [file pone.0090099.s005.docx]

**Table S1: Specific primers used for semiquantitative PCR**

| GENE | SEQUENCE (5’-3’)* | AMPLICON (bp) |
| --- | --- | --- |
| MMP-1 | F: GATGTGGAGTGCCTGATGTG | 200 |
|  | R: TGCTTGACCCTCAGAGACCT |  |
| MMP-2 | F: ACCCAGATGTGGCCAACTAC | 201 |
|  | R: TCATGATGTCTGCCTCTCCA |  |
| MMP-3 | F: CCTCAGGAAGCTTGAACCTG | 172 |
|  | R: GGGAAACCTAGGGTGTGGAT |  |
| MMP-7 | F: CAGATGTGGAGTGCCAGATG | 206 |
|  | R: TGTCAGCAGTTCCCCATACA |  |
| MMP-8 | F: CAACACCTCCGCAAATTACA | 201 |
|  | R: GGTTGGATAGGGTTGCTTGA |  |
| MMP-9 | F: AGTTCCCGGAGTGAGTTGAA | 195 |
|  | R: CTCCACTCCTCCCTTTCCTC |  |
| MMP-10 | F: GTGCTGTTGTGTCTGCCAGT | 200 |
|  | R: ACCTCCAACCCAAGGAACTT |  |
| MMP-12 | F: GCTTGCCAAATCCTGACAAT | 199 |
|  | R: TAAGCAGCTTCAATGCCAGA |  |
| MMP-13 | F: TAAGGAGCATGGCGACTTCT | 200 |
|  | R: GGTCCTTGGAGTGGTCAAGA |  |
| MMP-14 | F: CAAGCATTGGGTGTTTGATG | 199 |
|  | R: CTTGGGGTACTCGCTATCCA |  |
| GAPDH | F: TGGTATCGTGGAAGGACTCATGAC | 189 |
|  | R: ATGCCAGTGAGCTTCCCGTTCAGC |  |

#### *F: Forward R: reverse oligonucleotides

Sequence of oligonucleotides, forward (F) and reverse (R), for sqRT-PCR. The amplicon size is indicated in pair bases (pb). sqRT-PCR conditions were optimized for each primer-pair. Amplification reactions consisted of following steps: 95°C for 5 min, 25–30 cycles at 95°C for 30 sec; optimized annealing temperatures for 30 sec and 72°C for 10 min.
