# Supplementary material for: Gasdermin-B Promotes Invasion and Metastasis in Breast Cancer Cells
Source: PLoS One. 2014 Mar 27;9(3):e90099. doi: 10.1371/journal.pone.0090099 (PMC3967990; doi:10.1371/journal.pone.0090099)
Supplement: Table S2 — Primers used for qRT-PCR. Reference of Taqman and SybGreen assays used in qRT-PCR. The manufactures is indicated in the table. (DOCX) [file pone.0090099.s006.docx]

**Table S2: Primers used for qRT-PCR**

| GENE | SEQUENCE (5’-3’)* | Reference | Use |
| --- | --- | --- | --- |
| GSDMB | Hs00218565_m1 | Applied Biosystem | Taqman |
| GSDMB-1 | Hs00938445_m1 | Applied Biosystem | Taqman |
| GSDMB-2 | Hs00939390_m1 | Applied Biosystem | Taqman |
| GSDMB3&4 | Hs00940508_m1 | Applied Biosystem | Taqman |
| β2M | Hs00187842_m | Applied Biosystem | Taqman |
| mCherry | F: CTACGACGCTGAGGTCAAGA | SIGMA | SybrGreen |
|  | R: CGATGGTGTAGTCCTCGTTG |  |  |
| β-actin | F: CGGTTGGCCTTAGGGTTCA | SIGMA | SybrGreen |
|  | R: GTGGGCCGCTCTAGGCACCA |  |  |

#### *F: Forward R: reverse oligonucleotides

Reference of Taqman and SybGreen assays used in qRT-PCR. The manufactures is indicated in the table.
