# Supplementary material for: Gasdermin-B Promotes Invasion and Metastasis in Breast Cancer Cells
Source: PLoS One. 2014 Mar 27;9(3):e90099. doi: 10.1371/journal.pone.0090099 (PMC3967990; doi:10.1371/journal.pone.0090099)
Supplement: Protocol S1 — Identification of proteins by Mass sectrometry (MS). (DOCX) [file pone.0090099.s007.docx]

**Protocol S1: Identification of proteins by Mass sectrometry (MS)**

The indicated immunoprecipitated products were resolved by SDS-PAGE for MS analysis. Previously to tryptic digestion gels were stained with Coomassie blue and bands of interest from Coomassie-stained gels were excised manually, deposited in 96-well plates, and processed automatically in a Proteineer DP (Bruker Daltonics, Bremen, Germany). The digestion protocol was based on [1] with minor amendments: gel plugs were washed first with 50 mM ammonium bicarbonate and second with acetonitrile (ACN) prior to reduction with 10 mM DTT in 25 mM ammonium bicarbonate solution; and alkylation was carried out with 55 mM iodoacetamide in 50 mM ammonium bicarbonate solution. Gel pieces were then rinsed first with 50 mM ammonium bicarbonate and second with ACN, and were dried under a stream of nitrogen. Modified porcine trypsin (sequencing grade, Promega, Madison WI, USA), at a final concentration of 16 ng/μl in 25% ACN/50 mM ammonium bicarbonate solution, was added and the digestion took place at 37 °C for 6 h. The reaction was stopped by adding 0.5% trifluoroacetic acid (TFA) for peptide extraction. The eluted peptides were dried by speed-vacuum centrifugation and were resuspended in 4 μl of MALDI solution (70% ACN/0.1% TFA aqueous solution). A 0.8 μl aliquot of each peptide mixture was deposited onto a 386-well OptiTOF plate (Applied Biosystems, Framingham, MA, USA) and allowed to dry at room temperature. A 0.8 μl aliquot of matrix solution (3 mg/ml α-cyano-4-hydroxycinnamic acid in MALDI solution) was deposited onto the dried digest and allowed to dry at room temperature.

**References:**

[1] Shevchenko A, Wilm M, Vorm O, Mann M. (1996) Mass spectrometric sequencing of proteins silver-stained polyacrylamide gels. Anal Chem.;68(5):850-8.
