# Supplementary material for: Gasdermin-B Promotes Invasion and Metastasis in Breast Cancer Cells
Source: PLoS One. 2014 Mar 27;9(3):e90099. doi: 10.1371/journal.pone.0090099 (PMC3967990; doi:10.1371/journal.pone.0090099)
Supplement: Protocol S2 — MALDI peptide mass fingerprinting and MS/MS analysis. (DOCX) [file pone.0090099.s008.docx]

**Protocol S2:** **MALDI peptide mass fingerprinting and MS/MS analysis**

For MALDI TOF/TOF analysis, samples were automatically acquired in an ABI 4800 MALDI TOF/TOF mass spectrometer (Applied Biosystems) in positive ion reflector mode (the ion acceleration voltage was 25 kV to MS acquisition and 1 kV to MS/MS) and obtained spectra were stored into ABI 4000 Series Explorer Spot Set Manager. Peptide mass fingerprinting (PMF) and MS/MS fragment ion spectra were smoothed and corrected to zero baseline using routines embedded in ABI 4000 Series Explorer software v3.6. Each PMF spectrum was internally calibrated with mass signals of trypsin autolysis ions to reach a typical mass measurement accuracy of <25 ppm. Known trypsin and keratin mass signals, as well as potential sodium and potassium adducts (+21 Da and +39 Da) were removed from the peak list. To submit the combined PMF and MS/MS data to MASCOT version 2.2.04 software (Matrix Science, London, UK), GPS Explorer version 4.9 was used to search in a non-redundant NCBI protein database (NCBInr 20090406, <http://blast.ncbi.nlm.nih.gov/Blast.cgi>; 8,198,267 sequences, 2,824,199,726 residues).
